# Stochastic processes drive the dynamic assembly of bacterial communities in *Salix matsudana* afforested soils

**DOI:** 10.3389/fmicb.2024.1467813

**Published:** 2024-09-11

**Authors:** Can Wang, Abolfazl Masoudi, Min Wang, Yin Wang, Ze Zhang, Jingkun Cao, Jian Feng, Zhijun Yu, Jingze Liu

**Affiliations:** ^1^Hebei Key Laboratory of Animal Physiology, Biochemistry, and Molecular Biology, Hebei Collaborative Innovation Center for Eco-Environment, Hebei Research Center of the Basic Discipline of Cell Biology, Ministry of Education Key Laboratory of Molecular and Cellular Biology, College of Life Sciences, Hebei Normal University, Shijiazhuang, China; ^2^Department of Biological Sciences, University of Illinois, Chicago, IL, United States

**Keywords:** soil bacterial dynamics, temporal fluctuations, environmental influences, community structure analysis, soil pathogenic bacteria

## Abstract

**Introduction:**

This study investigates the dynamic shifts in soil bacterial communities within a *Salix matsudana* afforested ecosystem transitioning from agricultural land. Understanding the temporal variability in bacterial diversity and community structures is crucial for informing forest management and conservation strategies, particularly in regions undergoing afforestation.

**Methods:**

We employed high-throughput sequencing across three distinct months (August, September, and October) to analyze the temporal variability in bacterial community composition and diversity. Network analysis was utilized to identify keystone species and assess community stability under varying environmental conditions, including fluctuations in temperature and precipitation.

**Results:**

We uncover significant temporal variability in bacterial diversity and community structures, which are closely tied to fluctuations in temperature and precipitation. Our findings reveal the abundance of the dominant bacterial phyla, such as Actinobacteria and Proteobacteria, which did not change overall, highlighting the stability and resilience of the microbial community across seasonal transitions. Notably, the increasing similarity in community composition from August to October indicates a reduction in species turnover, likely driven by more homogeneous environmental conditions. Through comprehensive network analysis, we identify the pivotal role of keystone species, particularly the human pathogen *Nocardia*, in maintaining community stability under reduced soil moisture. The observed variations in community connectivity underscore the microbial community’s resilience and adaptability to seasonal shifts, with higher stability in August and October contrasting with the instability observed in September.

**Discussion:**

These results underscore the complex interplay between stochastic and deterministic processes in bacterial community assembly, significantly shaped by prevailing environmental conditions. The insights gained from this research have far-reaching implications for forestry management and conservation strategies, particularly in regions undergoing similar afforestation efforts.

## Introduction

Due to the progress of high-throughput sequencing techniques and bioinformatic analyses, we know a lot about the ecological drivers of bacterial diversity and community in the soil (Chu et al., 2020). Soil bacterial communities are highly dynamic and can undergo distinct community successions over minutes and years (Zhao et al., 2022). Even though space-for-time substitution methods could provide explanations for bacterial community temporal changes ranging from millennia to minutes, temporal dynamics in community structure and composition still receive much less attention than spatial distribution (Chu et al., 2020; Zhao et al., 2022). The temporal dynamics of humans (David et al., 2014; Gao et al., 2022) and agriculture microbiology (Larsen et al., 2023) have been thoroughly evaluated, but few high-resolution research studies have been conducted in afforested ecosystems despite evidence that afforestation is an essential determination of microbial structure (Wang M. et al., 2022). Bacterial structure exhibits temporal trends that can span from days (Zhao et al., 2022) to seasons (Wang et al., 2024a) and even years (Martinović et al., 2021). In a stable environment, soil bacterial community activities have inherent circadian rhythms (Zhao et al., 2022). By studying the shifts in bacterial community over time, we can gain insights into the composition and interactions of coexisting species, shedding light on the mechanisms behind community patterns (Barberán et al., 2012).

The temporal patterns of bacterial assembly may depend on the host species and exhibit idiosyncrasies, highlighting the necessity to understand microbiological assembly in various vegetation species. Although there is considerable knowledge regarding the initial microbiome assembly in grasses and agricultural crops, there needs to be more information on the initial microbiome of long-lived trees (Dove Nicholas et al., 2021). *Salix matsudana* is a wood species with a broad and natural distribution in North China (Ma et al., 2021). Based on the earlier publication, it has been established that the root-associated bacterial community assembly in rubber trees is primarily influenced by stochastic processes (Lan et al., 2023). Furthermore, the interplay of stochastic and selective factors determines the initial microbiome assembly during the first growing season of *Populus* (Dove Nicholas et al., 2021).

The dissimilarity among bacteria changed over time, eventually stabilizing when the sampling intervals were 1 year or more apart (Kivlin and Hawkes, 2020). These findings imply that finer-scale temporal sampling, precisely intervals of less than 6 months (such as several weeks to months), is crucial to accurately capture the intraspecific growth dynamics of bacteria in man-made forests. Microbial communities respond rapidly to environmental variations, often occurring at short temporal scales, such as days or hours. Therefore, conducting microbial community studies with high-resolution time series is crucial to capturing both swift changes resulting from species interactions and prolonged dynamics stemming from shifts in overall ecological conditions (Nyirabuhoro et al., 2021). Short-term climatic conditions, including daily maximum/minimum temperature and average precipitation, are identified as potential drivers of community changes (Martinović et al., 2021).

Despite the extensive research on the temporal dynamics of microbial communities in human and agricultural systems (Li et al., 2023; Ren et al., 2024; Wang et al., 2024a; Wang M. et al., 2022; Yang et al., 2023), there is a significant gap in understanding these dynamics within afforested ecosystems, particularly those involving long-lived tree species such as *S. matsudana.* While previous studies have primarily focused on spatial distribution or have employed space-for-time substitution methods, there is limited high-resolution temporal research in afforested regions, where soil bacterial communities may exhibit rapid and distinct changes in response to environmental conditions. The lack of fine-scale temporal studies, especially those capturing intervals shorter than 6 months, limits our ability to fully understand the intraspecific growth dynamics of bacteria in these man-made forests. Moreover, the role of soil pathogenic bacteria in influencing ecosystem stability and potential human health risks within afforested soils remains underexplored. Addressing this gap is crucial for advancing our knowledge of microbial community assembly and informing forest management and public health strategies in areas undergoing afforestation.

## Materials and methods

### Site description and sampling

Our sampling sites were located in the Xiong’an New Area (XNA) (39°3′25′′N, 116°7′28′′E), a state-level new area with both terrestrial and aquatic habitat in China, which was established in April 2017 by the Chinese government, and positioned as a “millennium plan and a national event” (Wang et al., 2020; Wang J. et al., 2022). More than 31,000 ha of afforestation had been planted with over 23 million nursery-grown vegetation in the XNA, raising its forest coverage rate from 11 to 34%.^1^ Soil samples were collected weekly from the root–zone areas of *S. matsudana* trees in three plots from August 1 to October 31, 2019. Stones and litter were removed from the samples before analysis. The 5-point sampling method was used at intervals of five to six trees, and five soil subsamples were thoroughly mixed to obtain one composite sample (Masoudi et al., 2018; Masoudi et al., 2020). The soil samples collected weekly corresponding to the month (August, September, and October) and week (the weeks of 1 August, 8 August, 15 August, 22 August, 29 August, 5 September, 12 September, 19 September, 26 September, 3 October, 10 October, 17 October, 24 October, and 31 October) data, marked as AugEW1, AugEW2, AugEW3, AugEW4, AugEW5, SepEW1, SepEW2, SepEW3, SepEW4, OctEW1, OctEW2, OctEW3, OctEW4, and OctEW5, respectively. In total, 42 soil samples were collected. Roots and pebbles were manually removed from the samples and then divided into three parts (Supplementary Figure S1). The first part was stored in a portable refrigerator (~ −20°C) (Foshan Aikai Electric Appliance Co., Ltd., Guangdong, China) to transport to the molecular ecological laboratory at Hebei Normal University and stored at −80°C. The second part was to determine soil water content, enzyme activity, and available nutrients stored at 4°C. The last part was dried at room temperature for 1 week to assess the soil’s physicochemical properties.

### Soil properties and enzyme activities

To measure properties such as pH, electrical conductivity (EC), total potassium (TK), total phosphorus (TP), total nitrogen (TN), total carbon (TC), and the carbon/nitrogen ratio (C/N), the soils were air-dried. In contrast, for properties such as ammonium nitrogen (NH_4_^+^-N), nitrate nitrogen (NO_3_^−^-N), soil water content (SW), and soil dehydrogenase (SDHA), the soils were stored at 4°C and analyzed promptly. Soil properties such as soil pH, EC, SW, NH_4_^+^-N, NO_3_^−^-N, TK, TP, and SDHA were determined according to our recent and previous publications (Wang et al., 2024a; Wang M. et al., 2022; Wu et al., 2023). TN, TC, and C/N were measured by the Vario Elemental Analyzer (Vario EL-3, Elementar, Germany). Daily high temperature (HT), daily low temperature (LT), and average precipitation (PP) data come from the China Weather Website^2^ (Yang et al., 2024).

### Amplicon sequencing

The soil DNA extraction, integrity, concentration, and purity were individually performed for each replication and conducted as described in our previous publications in recent months (Wang et al., 2024b; Wu et al., 2023). The bacterial 16S ribosomal RNA (rRNA) V3 − V4 region primer pairs 338F (5′-ACTCCTACGGGAGGCAGCAG-3′) and 806R (5′-GGACTACHVGGGTWTCTAAT-3′) primers were selected and amplified using ABI GeneAmp® 9,700 polymerase chain reaction (PCR) thermocycler (ABI, United States). The PCR reaction mixture including 4 μL 5 × TransStart® Fast Pfu buffer (Transgene Biotech, China), 2 μL 2.5 mM dNTPs, 0.8 μL each primer (5 μM), 0.4 μL TransStart® Fast Pfu polymerase, 0.2 μL 2 mg/mL BSA, 10 ng of template DNA, and ddH_2_O to a final volume of 20 μL (Wang et al., 2023). PCR amplification cycling conditions for 16S rRNA were as follows: initial denaturation at 95°C for 3 min, and then 27 cycles of denaturing at 95°C for 30 s, annealing at 55°C for 30 s, and extension at 72°C for 45 s, and single extension at 72°C for 10 min, and each sample was repeated three times (Yang et al., 2022). The triplicate amplicons were extracted from 2% agarose gel, purified by the PCR Clean-Up Kit (YuHua, China) according to the manufacturer’s instruction, and quantified using Qubit 4.0 (Thermo Fisher Scientific, United States). The library was constructed using the NEXTFLEX® Rapid DNA-Seq Kit (Bioo Scientific, United States) for 42 PCR-purified amplicons and then sequenced on the Illumina Miseq PE300 platform at Majorbio Bio-Pharm Technology Co., Ltd. (Shanghai, China).

We used multiple bacterial pathogen detection (MBPD) to excavate the animal, plant, and zoonotic pathogens based on 16S rRNA gene sequencing (Yang et al., 2023). Raw sequence data were deposited into the NCBI (National Center for Biotechnology Information) database with the accession number PRJNA714982.

### Data processing and bioinformatics analysis

Raw sequence data was quality-filtered and spliced according to the previous study from our laboratory (Wu et al., 2023). Then, the optimized sequences were clustered into operational taxonomic units (OTUs) with 97% similarity using UPARSE version 7.1 (Edgar, 2013), and the chimeric sequences were removed. To minimize the influences of sequencing depth for the data analysis about α-and β-diversity, the number of 16S rRNA gene sequences from every sample was rarefied to 30,892, and the average Good’s coverage was still higher than 95.97%, respectively. The taxonomy of each OTU representative sequence was evaluated using RDP Classifier version 2.2 (Wang et al., 2007) against the 16S rRNA gene database (Silva 138/16 s bacteria) using a confidence threshold of 0.7.

α-Diversity indices of Chao1, Shannon, phylogenetic diversity (PD), and Good’s coverage were calculated using Mothur version 1.30.1 (Schloss et al., 2009). We compared Mantel correlations between α-diversity indices (Shannon, Chao1, and PD) and environmental factors given geographic distance (999 permutations) by the “linkET” package in R software ver. 4.3.2 (Sunagawa et al., 2015). The relationships between microbial community similarity (Bray−Curtis and Jaccard distances) were measured using Spearman’s correlation by the “vegan” package (Xu et al., 2020). The null model was developed by Stegen et al. (2013) and used to quantify the contribution of different ecological processes to community assembly at the weekly and monthly temporal scales. β-Mean-nearest taxon distance (βMNTD) and β-nearest taxon index (βNTI) were calculated based on the null model by 999 randomizations (Stegen et al., 2012). |βNTI| > 2 and |βNTI| < 2 indicate the dominance of the deterministic and stochastic process, respectively. We further measured the ecological processes governing microbial community assembly using Bray–Curtis–based Raup–Crick (RC_bray_): variable selection (βNTI >2); dispersal limitation (|βNTI| < 2, RC_bray_ > 0.95); undominated (|βNTI| < 2, |RC_bray_| < 0.95); homogeneous dispersal (|βNTI| < 2, RC_bray_ < −0.95) and homogeneous selection (βNTI < −2; Wu et al., 2023; Bell et al., 2022). To evaluate the potential importance of stochastic processes to bacterial community assembly, we assessed the fit of the neutral community model for bacteria to predict the relationship between the frequency with which taxa occur and their abundance on the broader metacommunity (Sloan et al., 2006). The model index of “*m*” is the migration rate, i.e., a measure of dispersal limitation. Lower *m* values mean that bacterial communities are more limited in dispersal. The value *R*^2^ shows the overall fit to the neutral model (Chen and Wen, 2021; Probst et al., 2023). The ecological niche breadth of all OTUs in a community is used to obtain the community-level niche breadth and overlap using Levins’s niche breadth index (Levins, 1968) by the “spa” package. Furthermore, we calculated the occurrences of species generated that observed occurrence exceeding the upper and below 95% confidence interval were identified as generalists and specialists, respectively, and the remaining species were identified as habitat neutralists by the “EcolUtils” package (Li et al., 2022). To investigate the time variation on the bacterial community in the root zone soil of *S. matsudana*, the top 300 OTUs at each monthly time frame were used for the construction of co-occurrence, Spearman’s correlation coefficient (*r* > 0.9) and significance level (*p* < 0.05). The Fruchterman–Reingold layout algorithm visualized the network images in Gephi version 0.9.2. Moreover, the topological characteristics of the co-occurrence network, including average degree, average weighted degree, network diameter, graph density, modularity, average clustering coefficient, and average path length, were calculated in Gephi. Within-modular degree (*Zi*) and among-modular degree (*Pi*) were established to recognize bacterial keystones each month (Guimerà and Nunes Amaral, 2005). Topological roles of different nodes were divided into four categories: (I) peripheral nodes, nodes with *Zi* ≤ 2.5 and *Pi* ≤ 0.62; (II) module hubs, nodes with *Zi* > 2.5 and *Pi* ≤ 0.62; (III) network hubs, nodes with *Zi* > 2.5 and *Pi* > 0.62; and (IV) connectors: nodes with *Zi* ≤ 2.5 and *Pi* > 0.62 (Chen and Wen, 2021). We identified the network hubs, module hubs, and connectors as keystone taxa, which play essential roles in the microbial communities and potential functions. Based on the relative abundance of keystone OTUs, we classified into six categories following the previous studies (Wu et al., 2023): (1) Always abundant taxa (AAT), with a relative abundance ≥1% in all samples; (2) Conditionally abundant taxa (CAT), with a relative abundance ≥0.01% in all samples and ≥ 1% in some samples; (3) Always rare taxa (ART), with a relative abundance <0.01% in all samples; (4) Conditionally rare taxa (CRT), with a relative abundance <0.01% in some samples but never ≥1% in any sample; (5) Moderate taxa (MT), with a relative abundance between 0.01 and 1% in all samples; (6) Conditionally rare and abundant taxa (CRAT), with a relative abundance ranging from rare (<0.01%) to abundant (≥1%). Statistical data analyses were performed using one-way ANOVA and *t*-test to calculate the significance of differences using the JMP Pro version 16.0.0 (SAS Institute Inc., Cary, NC, United States) for Microsoft Windows. The visualization and calculations were accomplished using Adobe Illustrator 2021 (Adobe Systems Incorporated, San Jose, California, United States), RStudio ver. 2023.09.1, and GraphPad Prism version 9.4.0 (GraphPad Software, LaJolla, California, United States) for Microsoft Windows.

## Results and discussion

### Bacterial community relative abundance, α-diversity, and relationship with the environmental factors

**Figure 1:**
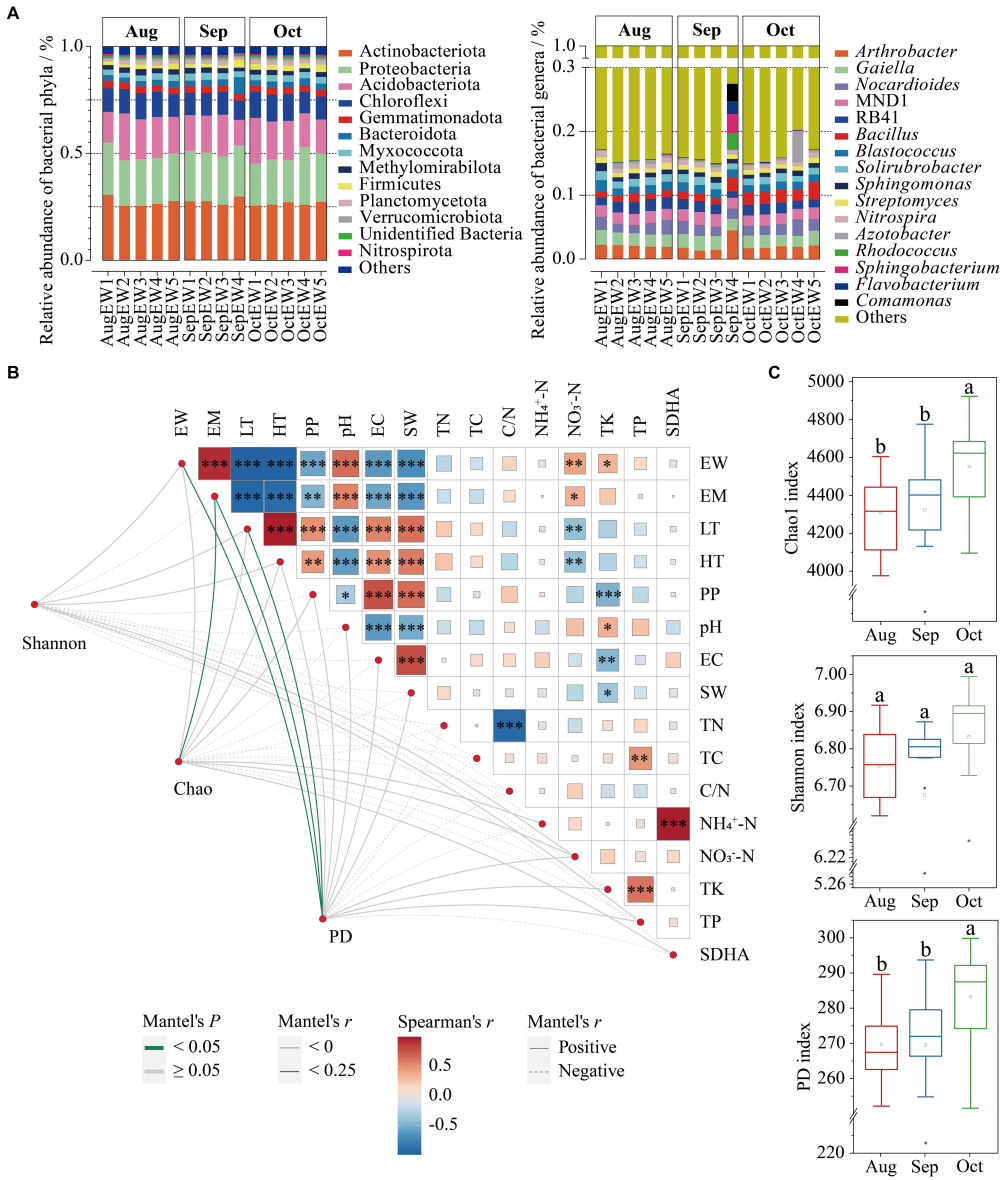
The relationship between α-diversity indices [Shannon, Chao1, and phylogenetic diversity (PD)] and environmental factors. The bacterial abundance distribution at the phyla and genera distribution weekly **(A)**. Mantel test showed the correlation between environmental factors (top node) and α-diversity indices (bottom node). The size of the square represents Spearman’s correlation coefficient (*r*) among environmental factors; *Significant difference with *p* < 0.05; ** Significant difference with *p* < 0.05; ***Significant difference with *p* < 0.05. Edges with positive and negative relationships are shown as solid and dashed. Edge color denotes the statistical significance based on 999 permutations, *p* < 0.05 and *p* ≥ 0.05 in green and gray colors, respectively **(B)**—the Chao1, Shannon, and PD indices from August to October **(C)**.

Among the primary bacterial phyla observed, Actinobacteria, Proteobacteria, Acidobacteria, and Chloroflexi exhibited relative abundances exceeding 10% (Figures 1A; Supplementary Table S1). Notably, Actinobacteria and Proteobacteria predominated in the soil of *S. matsudana*, aligning with our earlier finding from sampling plots featuring *Punus tabulaeformis*, *Sophora japonica*, and *Gingko biloba* afforestation in the XNA region (Wang et al., 2022; Wang M. et al., 2022). This consistency suggests that afforestation involving different tree species under similar environmental conditions does not markedly influence the bacterial community’s abundance in soil (Zhong et al., 2020). *Arthrobacter*, a resilient microbial genus tolerant to alkaline conditions, emerged as the predominant bacteria in the soil every week (Supplementary Table S2); known for its ability to thrive within a broad pH spectrum ranging from 7.0 to 12.0 with an optimal growth range at pH 7.0–8.0 (Lee et al., 2003), its prevalence in our experimental plot’s alkaline soil (Table 1) is unsurprising. The Firmicutes phylum and *Bacillus* genus demonstrated a significant positive correlation with each passing week and month (Mantel’s *r* = 0.149, 0.132, 0.275, and 0.233; *p* < 0.05; Supplementary Figures S2A,B), with their abundance showing a statistical increase every week (Supplementary Figure S2C). Notably, many families of Firmicutes that experienced growth under desiccation stress are recognized as spore-formers (Reimer et al., 2022). *Bacillus*, a genus within Firmicutes, exhibits remarkable adaptability to diverse environmental conditions, particularly under stressors such as warming and desiccation (Nicholson et al., 2000). This adaptability enables *Bacillus* to fulfill various ecological roles within the soil ecosystem and provide direct and indirect benefits to plants by aiding in nutrient acquisition (Liang et al., 2024; Saxena et al., 2020). The daily maximum /minimum temperature and average precipitation peak in July before gradually declining from August onward (Supplementary Figure S3). This pattern suggests that *Bacillus* can withstand fluctuating environmental stresses, as evidenced by its consistent presence in the soil across varying weekly conditions (Supplementary Table S2).

**Table 1:** The edaphic parameters in the root-zone soil of *Salix matsudana* for every week.

EP*	EW0801	EW0808	EW0815	EW0822	EW0829	EW0905	EW0912	EW0919	EW0926	EW1003	EW1010	EW1017	EW1024	EW1031
pH	8.12 ± 0.045^d^	8.16 ± 0.024^cd^	8.25 ± 0.017^ab^	8.17 ± 0.021^cd^	8.22 ± 0.015^bc^	8.20 ± 0.012^bc^	8.22 ± 0.008^bc^	8.21 ± 0.008^bc^	8.24 ± 0.024^ab^	8.22 ± 0.010^bc^	8.25 ± 0.018^ab^	8.28 ± 0.013^a^	8.24 ± 0.007^ab^	8.28 ± 0.014^a^
EC (mS/cm)	0.250 ± 0.010^a^	0.197 ± 0.008^b^	0.187 ± 0.005^bc^	0.176 ± 0.001^cde^	0.174 ± 0.007^cde^	0.165 ± 0.003^def^	0.163 ± 0.002^ef^	0.173 ± 0.002^cde^	0.168 ± 0.001^def^	0.158 ± 0.002^f^	0.163 ± 0.005^ef^	0.179 ± 0.003^cd^	0.168 ± 0.003^def^	0.162 ± 0.001^ef^
SW (%)	19.54 ± 0.355^a^	18.03 ± 0.674^a^	16.20 ± 0.392^b^	13.79 ± 0.462^c^	11.62 ± 0.143^def^	11.85 ± 0.267^def^	10.90 ± 0.424^f^	15.76 ± 0.463^b^	13.15 ± 0.480^cd^	10.62 ± 0.731^f^	11.57 ± 1.054^def^	11.49 ± 0.816^ef^	12.53 ± 0.532^cde^	11.33 ± 0.383^ef^
TN (g/kg)	0.93 ± 0.09^b^	1.49 ± 0.32^a^	1.06 ± 0.06^b^	1.09 ± 0.03^b^	1.01 ± 0.05^b^	1.06 ± 0.03^b^	1.05 ± 0.02^b^	1.14 ± 0.02^b^	1.14 ± 0.02^b^	1.19 ± 0.08^ab^	1.15 ± 0.17^b^	0.92 ± 0.02^b^	0.91 ± 0.04^b^	0.97 ± 0.08^b^
NH_4_^+^-N (mg/kg)	8.20 ± 0.43^ab^	6.13 ± 0.94^ab^	4.82 ± 0.56^b^	6.61 ± 0.83^ab^	8.79 ± 3.09^a^	7.06 ± 1.77^ab^	5.86 ± 1.32^ab^	6.02 ± 0.53^ab^	5.09 ± 0.102^ab^	8.91 ± 1.85^a^	5.91 ± 1.01^ab^	7.00 ± 0.90^ab^	7.23 ± 1.41^ab^	5.64 ± 1.01^ab^
NO_3_^—^N (mg/kg)	1.06 ± 0.26^b^	1.67 ± 0.62^b^	2.02 ± 1.06^b^	1.34 ± 0.65^b^	3.61 ± 1.01^ab^	2.25 ± 0.54^ab^	1.97 ± 0.16^b^	4.28 ± 1.83^ab^	2.77 ± 1.32^ab^	2.52 ± 1.00^ab^	2.11 ± 1.15^b^	5.52 ± 0.77^a^	5.42 ± 2.44^a^	2.77 ± 0.69^ab^
TC (g/kg)	19.73 ± 0.81^a^	19.94 ± 0.91^a^	20.00 ± 0.82^a^	19.95 ± 0.20^a^	19.88 ± 0.44^a^	19.71 ± 0.23^a^	20.38 ± 0.48^a^	19.85 ± 0.17^a^	19.45 ± 0.29^a^	19.29 ± 0.56^a^	19.18 ± 0.27^a^	19.54 ± 0.40^a^	19.72 ± 0.39^a^	19.97 ± 0.70^a^
C/N	21.45 ± 1.65^ab^	14.73 ± 3.15^f^	18.86 ± 0.57^abcde^	18.35 ± 0.77^abcdef^	19.79 ± 1.09^abcde^	18.58 ± 0.73^abcdef^	19.29 ± 0.13^abcde^	17.33 ± 0.44^cdef^	16.98 ± 0.41^def^	16.31 ± 0.77^ef^	17.56 ± 2.88^bcdef^	21.10 ± 0.32^abc^	21.57 ± 0.67^a^	20.62 ± 1.09^abcd^
SDHA (μg/d/g)	206.2 ± 9.37^ab^	161.5 ± 20.36^ab^	133.0 ± 12.31^b^	171.7 ± 18.00^ab^	218.9 ± 66.97^a^	181.5 ± 38.48^ab^	155.5 ± 28.68^ab^	159.1 ± 11.51^ab^	139.0 ± 2.01^ab^	221.6 ± 40.12^a^	156.7 ± 21.98^ab^	179.9 ± 19.60^ab^	185.3 ± 30.51^ab^	150.9 ± 22.03^ab^
TK (g/kg)	10.17 ± 2.85^b^	13.40 ± 0.83^a^	13.00 ± 0.35^a^	12.86 ± 0.30^a^	14.90 ± 0.58^a^	14.46 ± 0.56^a^	13.63 ± 0.34^a^	13.44 ± 0.25^a^	13.34 ± 0.44^a^	13.02 ± 0.22^a^	13.60 ± 0.44^a^	13.47 ± 0.82^a^	14.74 ± 0.31^a^	14.07 ± 0.50^a^
TP (g/kg)	0.610 ± 0.103^c^	0.748 ± 0.056^ab^	0.709 ± 0.042^abc^	0.686 ± 0.023^abc^	0.739 ± 0.045^abc^	0.654 ± 0.010^abc^	0.726 ± 0.015^abc^	0.690 ± 0.012^abc^	0.696 ± 0.034^abc^	0.646 ± 0.034^abc^	0.632 ± 0.059^bc^	0.774 ± 0.019^a^	0.686 ± 0.067^abc^	0.770 ± 0.038^a^

Values are means ± standard error with three replicates. Means with different letters indicate statistical significance (*p* < 0.05). EC: electrical conductivity, SW: soil water content, TN: total nitrogen, NH_4_^+^-N: ammonium nitrogen, NO_3_^−^-N: nitrate nitrogen, TC: total carbon, C/N: the carbon/nitrogen ratio, SDHA: soil dehydrogenase, TK: total potassium, TP: total phosphorus.*Edaphic Parameters.

The bacterial α-diversity, as measured by Chao1 (*F*_2, 41_ = 4.4052, *p* = 0.0188) and PD (*F*_2, 41_ = 4.8945, *p* = 0.0127) indices, exhibited statistically significant differences across each month, except the Shannon index (*F*_2, 41_ = 1.2101, *p* = 0.3091; Figure 1B). Specifically, bacterial richness and PD were notably higher in October. In contrast, Shannon diversity followed a similar trend but without significant differences. Remarkably, despite a decrease in daily temperatures from August to October, the diversity indices showed an increase (Figure 1C). This observation aligns with previous research indicating that reductions in bacterial diversity, driven by long-term climate warming in grassland soil due to experimental warming and concurrent decreases in soil moisture, led to a reduction in richness by 9.6% (Wu et al., 2022). The results indicate that lower daily temperatures could positively affect beneficial bacterial groups, particularly those in the Firmicutes phylum, within the soil surrounding *S. matsudana* trees in the XNA region.

### Variations of microbial community similarity and community assembly

**Figure 2:**
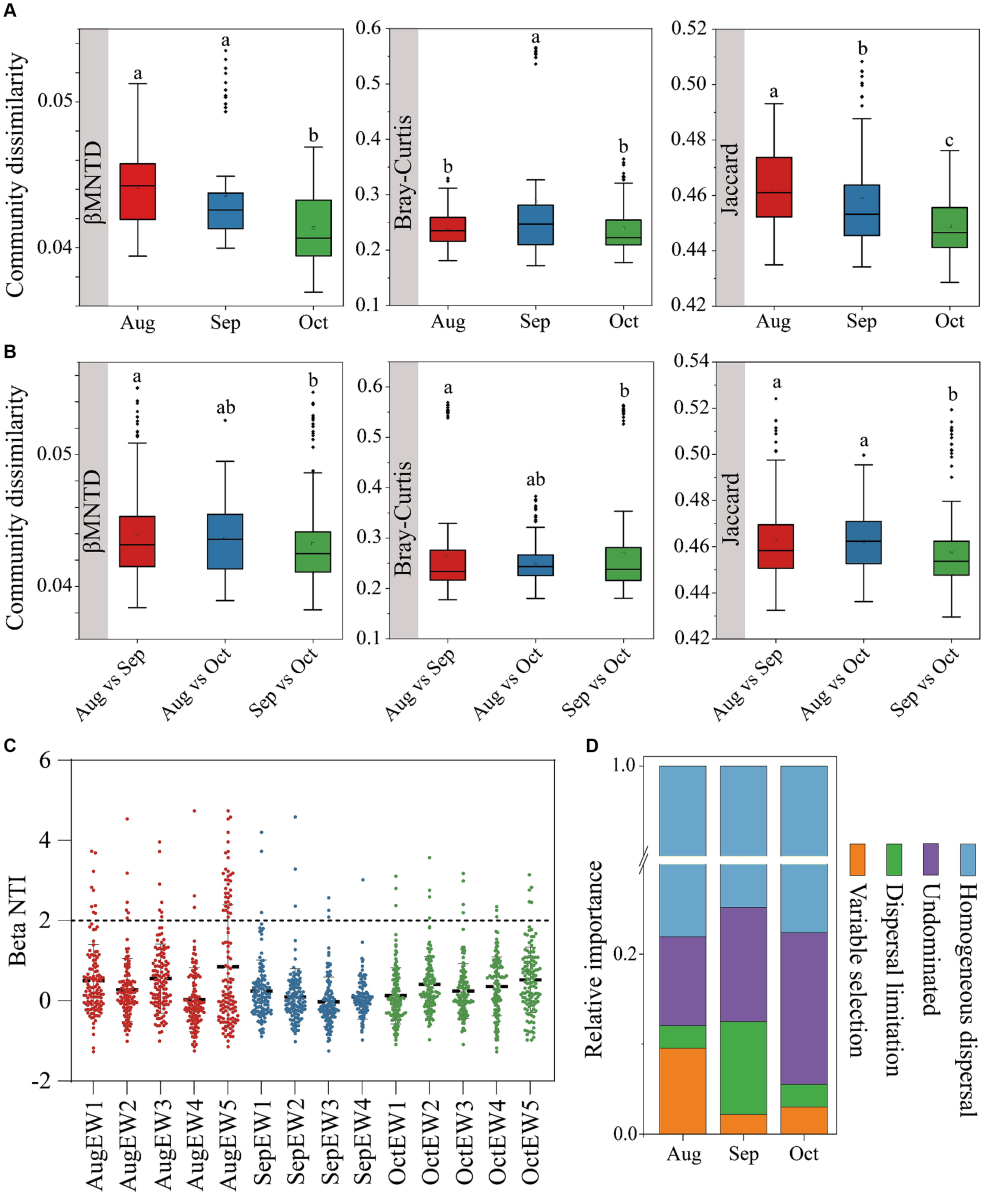
Bacterial community dissimilarities within **(A)** and between **(B)**, using βMNTD, Bray–Curtis, and Jaccard distance. The null model analysis reveals the assembly mechanism of the weekly temporal scale **(C)** bacterial communities and different ecological processes of the monthly temporal scale **(D)**.

Distinct variation patterns in the divergence and convergence of bacterial communities were observed within (Figure 2A) and between (Figure 2B) samples from August to October. Analyzing three dissimilarity metrics (Bray-Curtis, Jaccard, and βMNTD), our results suggest an increasing similarity in bacterial community compositions over this period. This heightened similarity points to a decrease in species turnover (Koleff et al., 2003), contrary to the typical temporal-decay pattern observed in general ecological knowledge (Shade et al., 2013; Xu et al., 2020). Two potential explanations may account for this phenomenon. Firstly, it is conceivable that the convergence of this community is a result of the decrease in daily temperature, which is consistent with research indicating that climate warming can lead to a divergent succession of microbial communities in grasslands (Guo et al., 2018). Secondly, previous studies have shown that bacterial communities tend to become more divergent over short periods (less than 6 months) and more convergent over more extended periods (more than 25 weeks), which directional change over short time intervals may be attributed to the high adaptability of different bacterial taxa to environmental variations, such as seasonal changes in temperature (Nyirabuhoro et al., 2021; Wang et al., 2024a).

Insights into community assembly mechanisms can be gleaned from β diversity results (Bell et al., 2022; Zhou et al., 2014). Stochastic processes such as birth, death, and dispersal events played a significant role in shaping bacterial communities, as evidenced by |βNTI| values predominantly less than 2 (Figure 2C). These values suggest that bacteria could proliferate and disperse more freely under lower selective pressure (Mo et al., 2021). Contribution to bacterial community assemblies from August to October ranged from 77.62 to 78.10% (Figure 2D), indicating that homogeneous dispersal was the primary process, signifying a robust diffusion effect among bacterial species. This is partly attributed to the tendency for communities in heterogeneous environmental patches to homogenize, leading to community convergence and a decrease in bacterial community β-diversity (Wu et al., 2023). Our findings revealed percentages of homogenous dispersal at 78.10, 74.80, and 77.62% in August, September, and October, respectively. Intuitively and mechanistically, an increase in dispersal is predicted to decrease β diversity because local communities become more similar in species composition as species colonize all patches (Grainger and Gilbert, 2016). Additionally, microbes may disperse to areas with greater water availability, such as water-filled soil pores acting as refugia (Allison, 2023).

### Niche overlap and breadth

**Figure 3:**
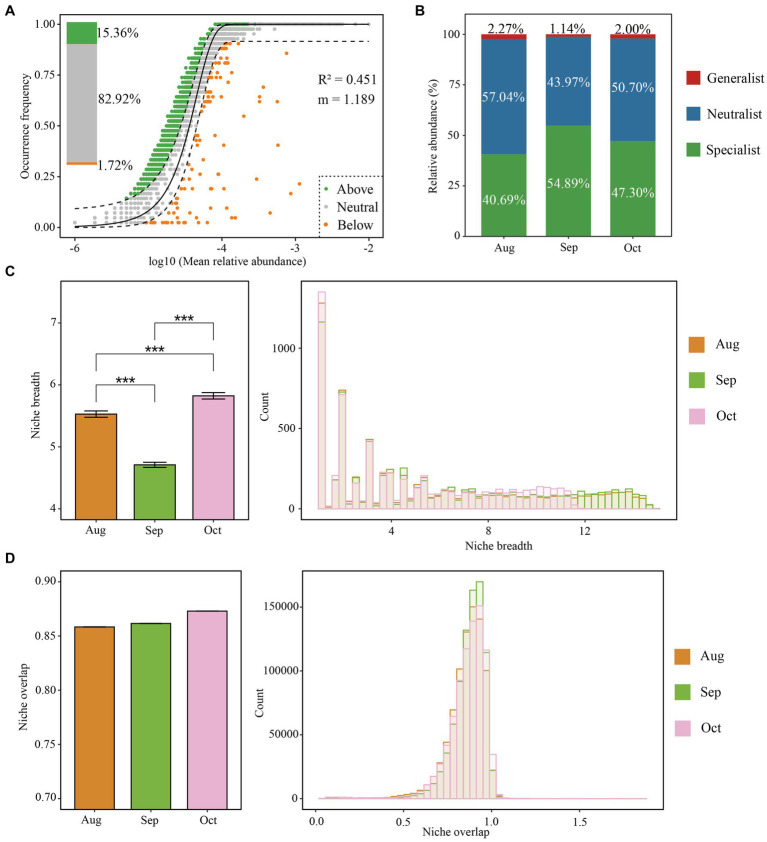
Assembly mechanisms of the bacterial community of the root–zone soil of *Salix matsudana* from August to October. The neutral community model (NCM) of community assembly **(A)**. The solid black line indicates the perfect fit of NCM, and the dashed line represents the 95% confidence interval around the model predictions. Operational taxonomic units (OTUs) in green, gray, and yellow points represent above, within, and below the frequency range. The OTU relative abundance of bacterial generalist, specialist, and the neutralist **(B)**. Niche breadth and overlap **(C,D)** for bacterial communities.

The Sloan neutral model reveals that bacterial communities were primarily shaped by stochastic processes from August to October. This is supported by a correlation coefficient (R^2^) of 0.451 and an estimated migration rate (*m*) of 1.189, indicating stochastic influences. A substantial portion of bacterial OTUs, amounting to 82.92%, aligned with the expectations of the neutral community model, further confirming the impact of stochastic processes on bacterial dynamics. This finding was consistent with results from the null model analysis (Figure 3A). The *m* values for August, September, and October were 1.187, 1.255, and 1.299, respectively (Supplementary Figures S4A–C), suggesting relatively low dispersal limitations on bacterial communities during this period. Consequently, we computed habitat niche breadth overlap and identified generalists, specialists, and neutralists (Figure 3B). The results indicate that the ecological niche breadths of soil bacteria differ significantly across the months, with October displaying the broadest niche, followed by August, and then September (October > August > September). (Figure 3C). This trend indicates that the soil bacteria adapt their ecological strategies differently across these months, possibly due to changes in environmental conditions or resource availability, given the previously established understanding that microbes in environments with wider niche breadths experience less habitat stress (Li et al., 2022). We identified that the soil in September harbored the more significant number of habitat specialists, with fewer generalists and neutralists compared to October and August (Figure 3B). Particularly, bacterial taxa that are habitat generalists seem to be primarily affected by spatial factors, while those that are specialists tend to be more influenced by environmental conditions. This indicates that random processes mainly govern the distribution of generalists, whereas specialists are more often shaped by specific ecological niches during the formation of communities (Li et al., 2022; Mo et al., 2021).

Furthermore, the data indicate a noticeable increase in niche overlap (Figure 3D). This trend, coupled with reduced dispersal limitations (Supplementary Figure S4) and the lessened significance of variable selection as depicted (Figure 2D), from August to October, supports the hypothesis that older afforestation efforts might lead to a decrease in habitat diversity. In addition, temperature variations affect microbial dynamics, where the slower bacterial growth rates observed during the cooler monthly temperatures contribute to a more uniform performance across species compared to the variations seen at higher temperatures (Zhao et al., 2023). This uniformity in growth conditions likely fosters greater similarity among bacterial populations in soils that have undergone afforestation, suggesting a convergence in community characteristics over time due to stable, less variable environmental conditions.

### Network complexity in monthly temporal scale

**Figure 4:**
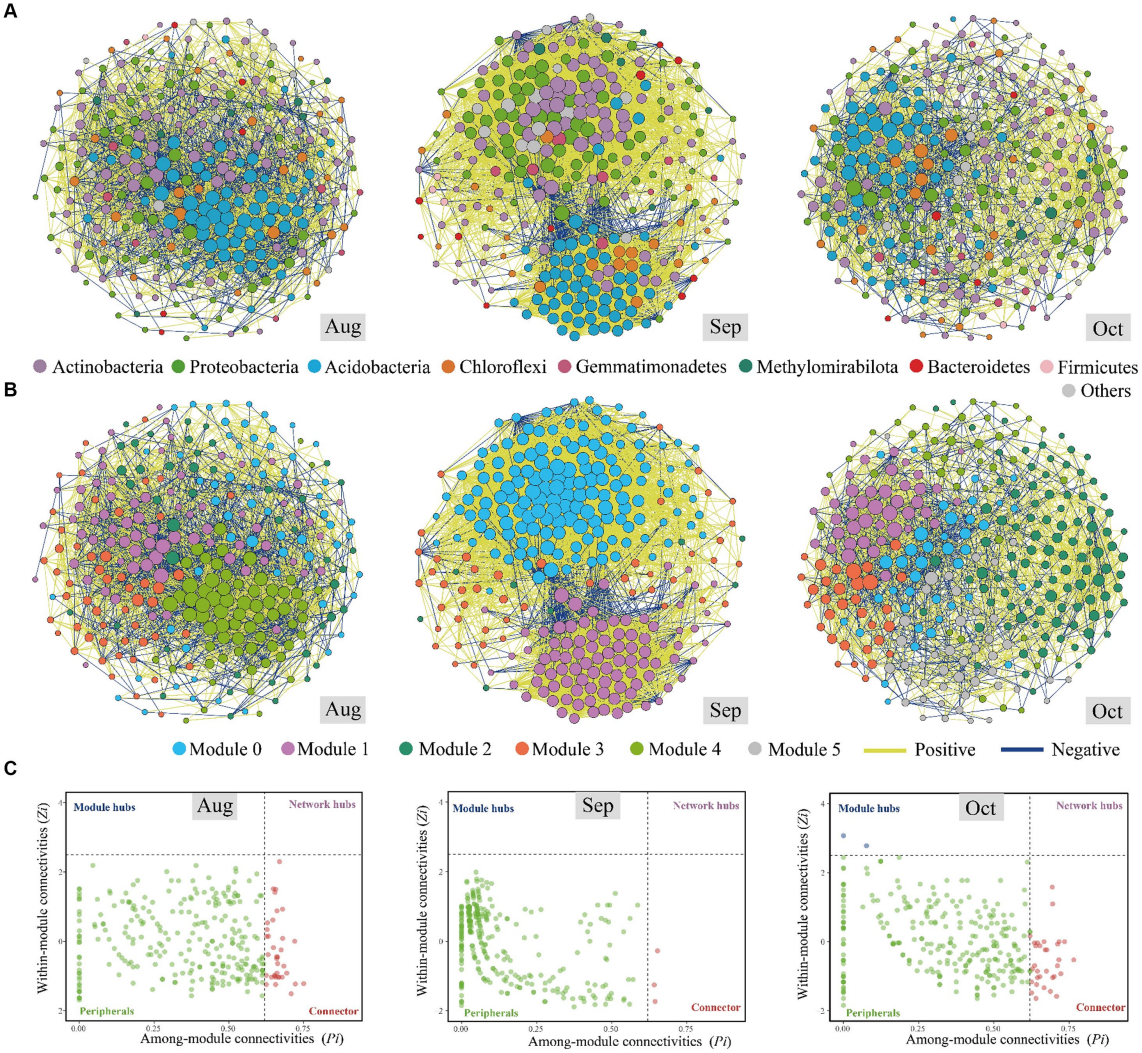
Co-occurrence networks of the soil bacterial communities of *Salix matsudana* in August, September, and October based on pairwise Spearman’s correlations between operational taxonomic units (OTUs). The lines in yellow and blue between each pair of nodes represent positive and negative interactions with strong (Spearman’s correlation coefficient*, r* > 0.9 or *r* < −0.9) and statistical (*p* < 0.05) correlation. The size of each node is proportional to the number of connections (i.e., degree). The nodes were colored according to the top eight phyla in relative abundance, and the rest of the bacterial phyla were grouped into “others” **(A)**. The nodes were colored according to various modularity classes in August, September, and October **(B)**. The *Zi–Pi* plots of soil bacteria based on OTU level **(C)**.

Bacterial network analysis unveils intricate interaction dynamic and ecological processes extending beyond bacterial communities’ mere composition and richness. Comparatively, the co-occurrence network in September displayed greater complexity than in August and October (Figures 4A,B; Table 2). Generally, a more intricate network structure suggests enhanced efficiency in resource transfer and more stable coexistence patterns (Chen and Wen, 2021), implying that bacterial communities in September exhibited higher resilience to environmental disturbance. In our investigation, the prevalence of positive associations in September (92.53%) surpassed that of August (62.34%) and October (65.77%), indicating the heightened significance of positive effects such as mutualism and/or syntropy, wherein two species exchange metabolic products to their mutual benefit, compared to the adverse impacts, such as predator–prey relationships, host–parasite relationships, and/or competition among microbiomes (Chen and Wen, 2021). This observation finds further support in the significantly lower niche breadth in September (Figure 3C), suggesting that environmental factors exerted intense selective pressures during that month (Lin et al., 2021). Our speculation suggests that bacterial communities become more resilient under more significant environmental stresses. When considering the degree, closeness, betweenness, and eigenvector centrality features of the co-occurrence network (Supplementary Figure S5), our findings indicate that the stress experienced in September significantly altered the bacterial communities, which were then adapted by October. We evaluated the potential topological roles of OTUs within the co-occurrence network, drawing from the findings of *Zi* and *Pi* (Ji et al., 2021; Figure 4C).

**Table 2:** Topological characteristics of bacterial co-occurrence networks.

	August	September	October
Nodes	298	300	299
Edges	3,837	8,193	2,483
Positive connections	2,392 (62.34%)	7,581 (92.53%)	1,633 (65.77%)
Negative connections	1,445 (37.66%)	612 (7.47%)	850 (34.23%)
Average degree	25.752	54.62	16.609
Average weighted degree	17.875	39.678	11.355
Network diameter	6.0	5.0	6.0
Graph density	0.087	0.183	0.056
Modularity	0.350	0.419	0.454
Average clustering coefficient	0.43	0.613	0.342
Average path length	2.486	2.161	2.733

Most nodes in the bacterial network were classified as peripherals, with no nodes identified as network hubs, which serve as connectors and module hubs. In August, September, and October, 36, 3, and 34 OTUs were recognized as keystone species. The relatively high number of connectors (25 OTUs) in August indicates a well-integrated network, with numerous OTUs playing crucial roles in linking different modules. This suggests that the bacterial community in August exhibited a complex structure with a high degree of connectivity, potentially facilitating efficient interactions and exchanges across the community (Wu et al., 2023). The sharp decrease in the number of connectors (3 OTUs) in September reflects a significant reduction in network integration. This indicates that the microbial community became less interconnected, possibly due to increased environmental stress or selective pressures. Such a reduction in connectivity could lead to decreased stability and resilience of the microbial community, as fewer OTUs act as bridges between modules. The substantial increase in the number of connectors (32 OTUs) in October suggests a reestablishment or even enhancement of network connectivity. This indicates a more integrated and potentially more stable microbial community compared to September. The rise in the number of connectors could be a response to changing environmental conditions, reflecting the community’s adaptation to become more resilient and interconnected (Chen and Wen, 2021). The fluctuations in the number of connectors reflect the microbial community’s dynamic adaptation to seasonal environmental shifts. August’s relatively high connectivity, September’s decline, and October’s recovery indicate that the community is responsive to changes in temperature and moisture conditions. The higher number of connectors in August and October suggests periods of more excellent stability and resilience, with the microbial community being better equipped to maintain its functions despite environmental changes. September’s low number of connectors indicates a period of instability or stress. The variations align with the significant impact of environmental conditions (e.g., temperature and precipitation) on community assembly processes, as previously discussed. The microbial community’s structure changes in response to these conditions, impacting the roles and connectivity of different OTUs (Supplementary Table S3).

Actinobacteria, known for its degrading abilities and adaptive physiological traits enabling survival during drought (Allison, 2023), constituted 27.78% (10 OTUs), 66.67% (2 OTUs), and 29.41% (10 OTUs) of the bacterial composition from August to October. This suggests that *S. matsudana* allocates more carbon below ground to acquire water, responding to drought conditions. However, if the productivity of *S. matsudana* declines due to water limitations, reallocation may not sustain carbon inputs to the soil. Thus, field and laboratory experiments employing a hierarchical, multiscale approach are needed to address knowledge gaps concerning soil carbon balance under drought. In September, the predominant keystone species were from the Actinobacteria and Chloroflexi groups, reflecting similar bacterial communities found on sorghum leaves affected by drought stress (Gao et al., 2022). With a reduction in soil moisture from August to October (Table 1) and a notable decrease in precipitation in September (Supplementary Figure S3), *Nocardia* as a keystone species was likely a significant factor influencing these environmental changes. Remarkably, the genus *Nocardia*, known for causing mycetoma in humans and animals, was prevalent in brown soil samples, especially those that were exceedingly dry (Ishikawa et al., 2004; Valero-Guillén and Martín-Luengo, 1984).

### Soil pathogenic bacteria

**Figure 5:**
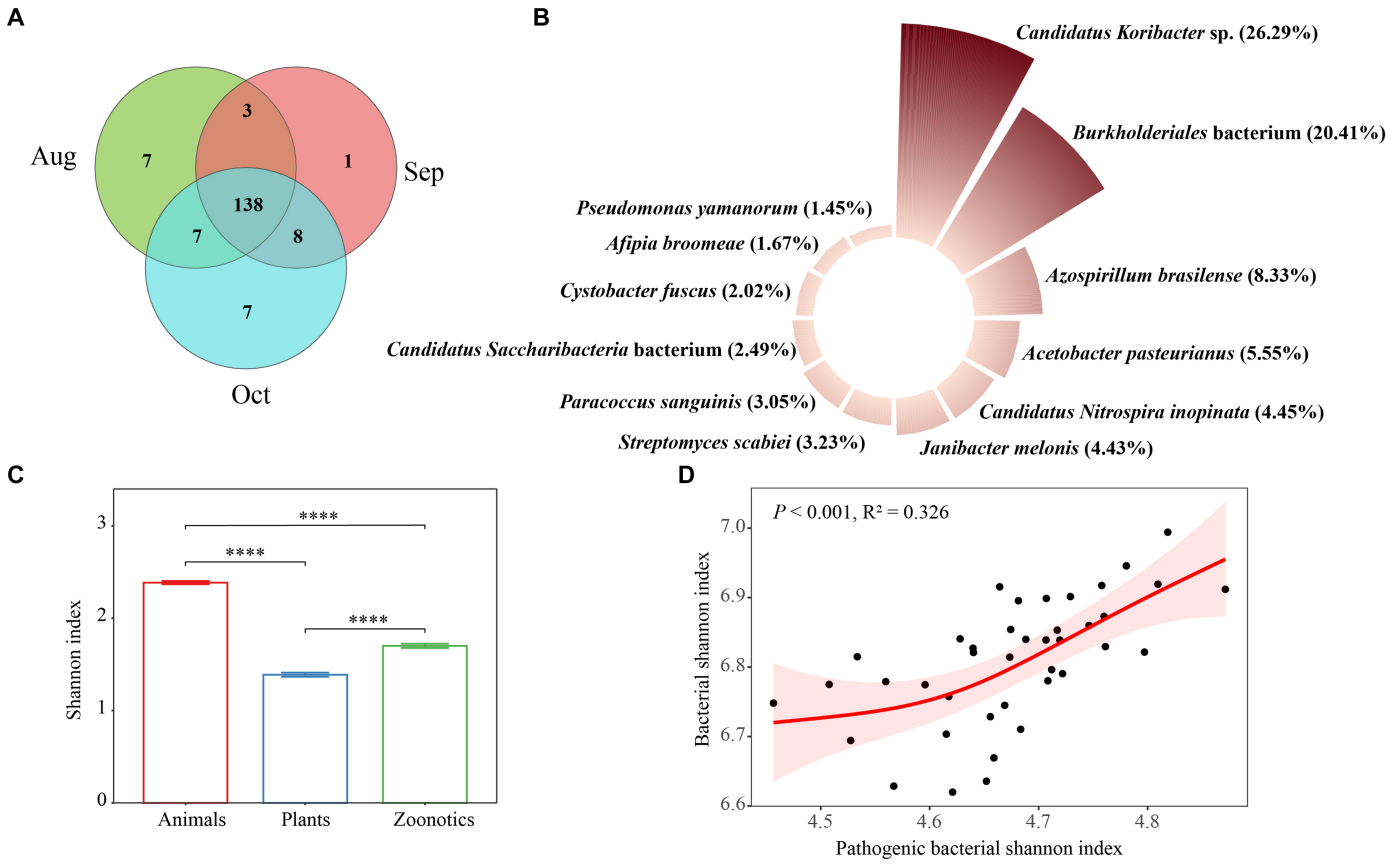
Venn results for soil pathogenic bacteria of *Salix matsudana* in August, September, and October **(A)**. Main species **(B)**, Shannon index **(C)** of soil pathogenic bacteria, and the relationship **(D)** shown by the generalized additive model (GAM) between pathogenic bacterial Shannon index and bacterial Shannon index in study area *S. matsudana* overall these 3 months.

Soil pathogenic bacteria are significant biological contaminations in terrestrial environments, posing potential threats to soil ecosystems and human health (Li et al., 2023). A Venn diagram revealed that the core soil pathogenic bacterial taxa across August, September, and October consisted of 138 species, accounting for 80.7% of the total, indicating that most taxa are common pathogens across these 3 months (Figure 5A). We also evaluated the monthly variations in the abundance of soil pathogenic bacteria, as depicted in Supplementary Figure S6A and summarized in Figure 5B. The primary pathogens identified were *Candidatus Koribacter* sp., *Burkholderiales* bacterium, and *Azospirillum brasilense*, each demonstrating monthly fluctuations in abundance. A previous study has emphasized that environmental conditions, such as temperature and humidity, vary by season and significantly impact the prevalence of these pathogenic bacteria in soil, thus influencing their occurrence (Wang C. et al., 2022). The *Candidatus Koribacter* genus refers to a group of bacteria that are part of the Acidobacteria phylum commonly found in soil. These bacteria are known for their role in soil ecosystems, particularly in the processes related to soil nitrogen cycling, including the reduction of nitrate, nitrite, and nitric oxide (Lacerda-Júnior et al., 2019) and carbon degradation processes such as the breakdown of cellulose hemicellulose, and chitin (Zhang et al., 2020). The species in the Burkholderiales order exhibit a remarkable range of metabolic capabilities and ecological roles, making them of great interest across multiple fields, including microbiology, environmental science, and biotechnology. However, there are also pathogenic species within this order that are known to cause infections in humans, especially in those with compromised immune systems. These infections can be challenging to treat due to their resistance to multiple antibiotics (Morya et al., 2020). In our research, the precise function of the *Burkholderiales* bacterium (NCBI: txid469610) remains undetermined and warrants additional study, especially given its high prevalence in the afforested regions of the urban XNA region. The Shannon index, a measure of biodiversity, showed that animal pathogens had a significantly higher index (*p* < 0.001) compared to zoonotic pathogens, which in turn were higher than plant pathogens, following the order: Animal > zoonotic > plant (Supplementary Figure S6B) monthly and overall (Figure 5C). Our findings indicate that the robust, nonlinear association between the Shannon index of pathogenic bacteria with overall bacterial Shannon diversity, suggesting that the relationship between these two features is non-monotonic (Figure 5D). Thus, a single measure may not comprehensively account for the relationship between the composition and diversity of soil bacterial pathogens and, consequently, the overall health. However, we anticipate that future research in the XNA region will enhance our understanding of the risks soil pathogens pose to animal and human health. It has been previously shown that soil bacterial diversity is a critical factor in managing the prevalence of pathogenic bacteria, such as *Escherichia coli* (Van Elsas et al., 2012), plant pathogens at the wheat seedling stage (Cui et al., 2023), and the pathogens of reductive soil disinfestation in the greenhouse (Duan et al., 2025). The proliferation of bacteria in the soil of *S. matsudana* has led to an increase in bacterial pathogens, posing health risks to people utilizing urban green space.

### Ecological and environmental implications

Our study reveals that soil bacterial communities in afforested *S. matsudana* ecosystems experience significant temporal shifts, driven by environmental changes such as temperature and precipitation. These shifts extend beyond academic interest, carrying important ecological implications. For example, the observed convergence in bacterial community composition from August to October indicates that as environmental conditions become more uniform, species turnover decreases. This suggests that afforested ecosystems may become increasingly resilient to environmental fluctuations as bacterial communities stabilize and adapt to consistent conditions (Huang et al., 2024; Ren et al., 2024). Furthermore, our study emphasizes the role of stochastic processes in shaping these bacterial communities. This finding highlights the need to consider both deterministic and stochastic factors in ecosystem management, as their interplay can significantly impact the stability and functioning of the ecosystem. This is vital for maintaining soil health, supporting plant growth, and preserving biodiversity. From an environmental management standpoint, these insights can guide strategies for afforestation and reforestation projects, especially in areas undergoing land-use change. Understanding how microbial communities respond to afforestation can help predict the long-term sustainability of these ecosystems and inform the design of interventions that bolster their resilience to climate change (Luo et al., 2023). Additionally, our findings have public health implications due to the presence of pathogenic bacteria in the soil. Monitoring these microbial communities can help assess potential health risks, particularly in urban areas where afforested lands are increasingly utilized for recreational purposes.

## Conclusion

This study highlights considerable fluctuations over time in bacterial diversity and community configuration. These fluctuations are significantly influenced by environmental factors such as temperature and precipitation, reinforcing the core ecological concept that environmental conditions are vital in forming bacterial community assembly. Our findings reveal the abundance of the dominant bacterial phyla, such as Actinobacteria and Proteobacteria did not change overall, highlighting the stability and resilience of the microbial community across seasonal transitions. Such adaptability demonstrates soil bacteria’s resilience and functional flexibility, essential for maintaining ecosystem health. The observed increase in community similarity from August to October implies a reduction in species turnover, likely due to more consistent or unifying environmental conditions. This observation is crucial for understanding the potential responses of bacterial communities to long-term ecological changes induced by climate change or land-use alterations. The results show that stochastic processes play critical roles in shaping the assembly of bacterial communities. This interplay adds a layer of complexity to bacterial dynamics, as random events and environmental pressures collectively influence community structure. The findings from this study are invaluable for forestry management and conservation efforts. Building on the findings of this study, we intend to extend the temporal scope of our observations to capture long-term trends in bacterial community assembly and their responses to seasonal and annual climatic variations. In light of the public health concerns identified, we will also investigate the dynamics of soil-borne pathogens more comprehensively, focusing on how afforestation and changing environmental conditions influence their prevalence and associated risks to human and animal health. Finally, we aim to collaborate with forestry managers and public health experts to translate our scientific findings into practical recommendations for forest management and urban planning, particularly in regions undergoing afforestation.

## Data availability statement

The datasets presented in this study can be found in online repositories. The names of the repository/repositories and accession number(s) can be found in the article/Supplementary material.

## Author contributions

CW: Conceptualization, Data curation, Formal analysis, Methodology, Software, Validation, Writing – original draft, Funding acquisition. AM: Conceptualization, Data curation, Formal analysis, Methodology, Software, Supervision, Validation, Writing – original draft. MW: Conceptualization, Validation, Writing – original draft. YW: Conceptualization, Software, Writing – original draft. ZZ: Conceptualization, Data curation, Writing – original draft. JC: Conceptualization, Writing – original draft. JF: Conceptualization, Writing – original draft. ZY: Conceptualization, Supervision, Writing – review & editing. JL: Funding acquisition, Project administration, Supervision, Writing – review & editing.

## Footnotes

^1^ https://english.news.cn/20230402/b62da58ca17b473ba7c43a03fc868465/c.html

^2^ http://www.weather.com.cn/weather/101091204.shtml
