# Supplementary material for: Stochastic processes drive the dynamic assembly of bacterial communities in Salix matsudana afforested soils
Source: Front Microbiol. 2024 Sep 11;15:1467813. doi: 10.3389/fmicb.2024.1467813 (PMC11422207; doi:10.3389/fmicb.2024.1467813)
Supplement: Supplementary file 1 [file Data_Sheet_1.pdf]

Supplementary File for

Stochastic processes drive the dynamic assembly of bacterial communities in *Salix Matsudana* afforested soils

Can Wang†[1], Abolfazl Masoudi†[2], Min Wang[1], Yin Wang[1], Ze Zhang[1], Jingkun Cao[1], Jian Feng[1], Zhijun Yu[*1], Jingze Liu[*1]

[1]Hebei Key Laboratory of Animal Physiology, Biochemistry, and Molecular Biology, Hebei Collaborative Innovation Center for Eco-Environment, Hebei Research Center of the Basic Discipline of Cell Biology, Ministry of Education Key Laboratory of Molecular and Cellular Biology, College of Life Sciences, Hebei Normal University, Shijiazhuang, Hebei 050024, PR China

[2]Department of Biological Sciences, University of Illinois, Chicago, Illinois, United States of America

† These authors contributed equally

Can Wang: wangcan@hebtu.edu.cn

Abolfazl Masoudi: amasou7@uic.edu

Min Wang: wangmin0811@stu.hebtu.edu.cn

Yin Wang: wangyin@hebtu.edu.cn

Ze Zhang: zhangze1666@163.com

Jingkun Cao: caojingkun@stu.hebtu.edu.cn

Jian Feng: fengjian@hebeu.edu.cn

*Corresponding Authors: Tel/fax: +86 311 80787519, yuzhijun@hebtu.edu.cn (ZY), liujingze@hebtu.edu.cn (JL)

Fig. S1 Summary schematic diagram of experimental design.

Fig. S2 Mantel test result about soil properties between the relative abundance of phyla (A) and genus (B).

Fig. S3 Daily maximum / minimum temperature and average precipitation of every month in 2019.

Fig. S4 NCM of August (A), September (B), and October (C).

Fig. S5 Co-occurrence network of microbial OTUs based on node features of the network.

Fig. S6 Main species of soil pathogenic bacteria in the study area (A), Shannon index (B), and the relationship (C) shown by the generalized additive model (GAM) between pathogenic bacterial Shannon index and bacterial Shannon index in August, September, and October.

Table S1. The abundance of the top ten bacterial phyla in the soil rhizosphere of *Salix matsudana* for every week (Mean ± SE).

Table S2. The abundance of the top ten bacterial genera in the soil rhizosphere of *Salix matsudana* for every week (Mean ± SE).

Table S3. Lists of keystone taxa in co-occurrence network of bacteria.

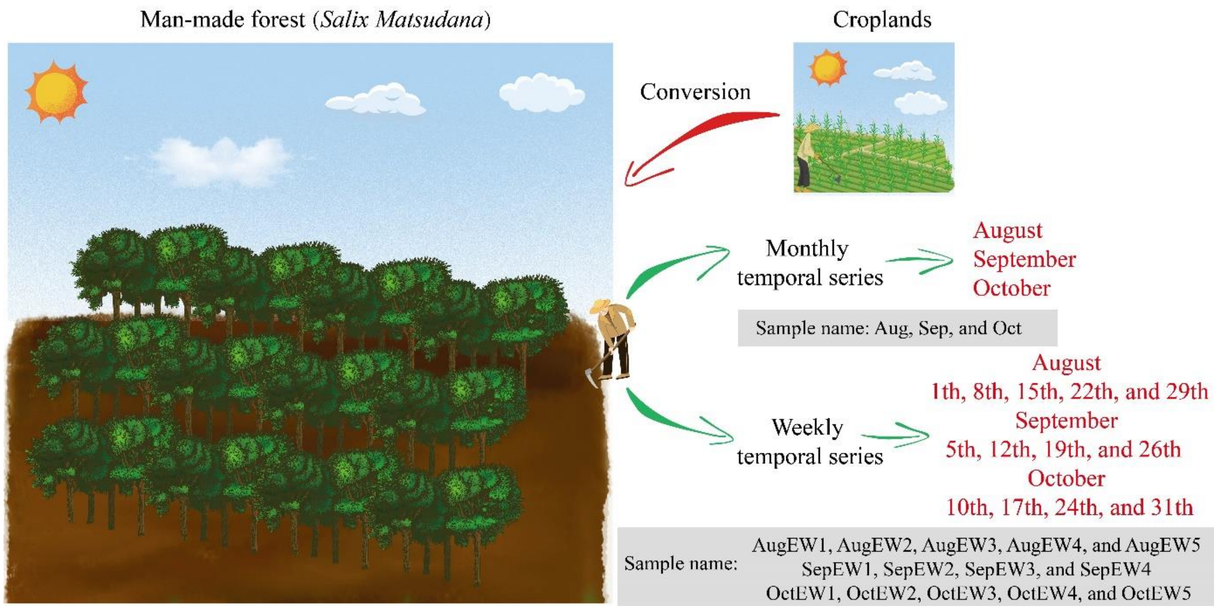

Fig. S1 Summary schematic diagram of experimental design.

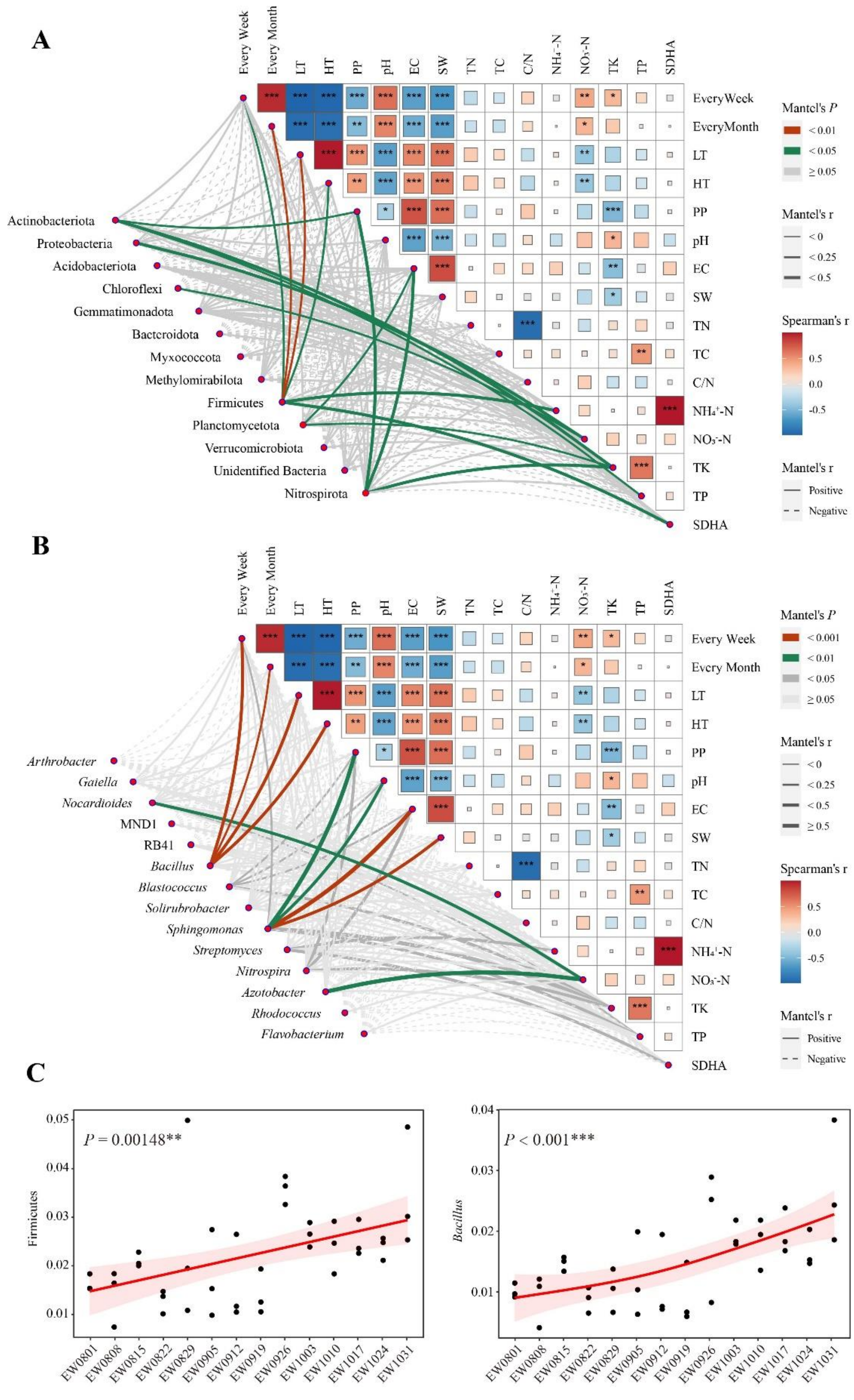

Fig. S2 Mantle test result about soil properties between the relative abundance of phyla (A) and genus (B).

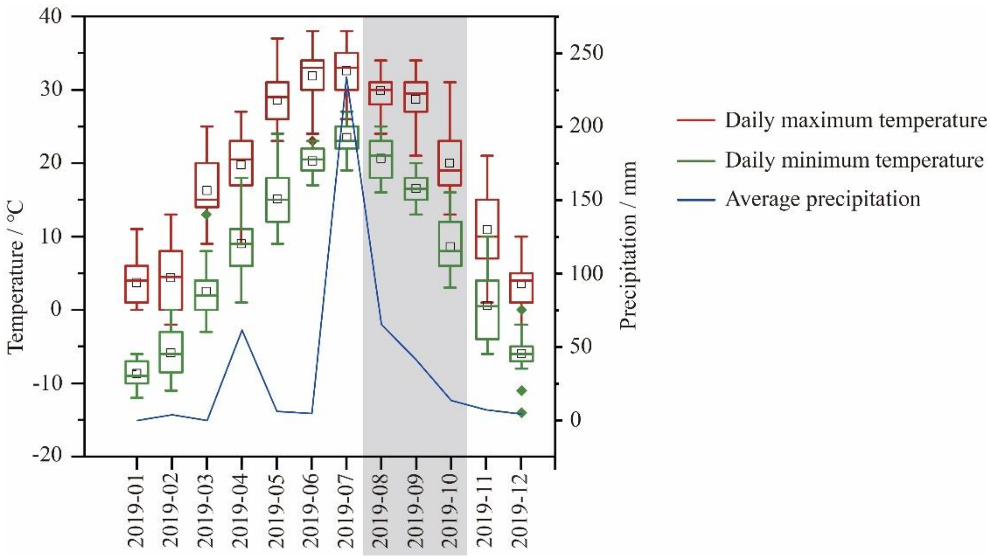

Fig. S3 Daily maximum / minimum temperature and average precipitation of every month in 2019.

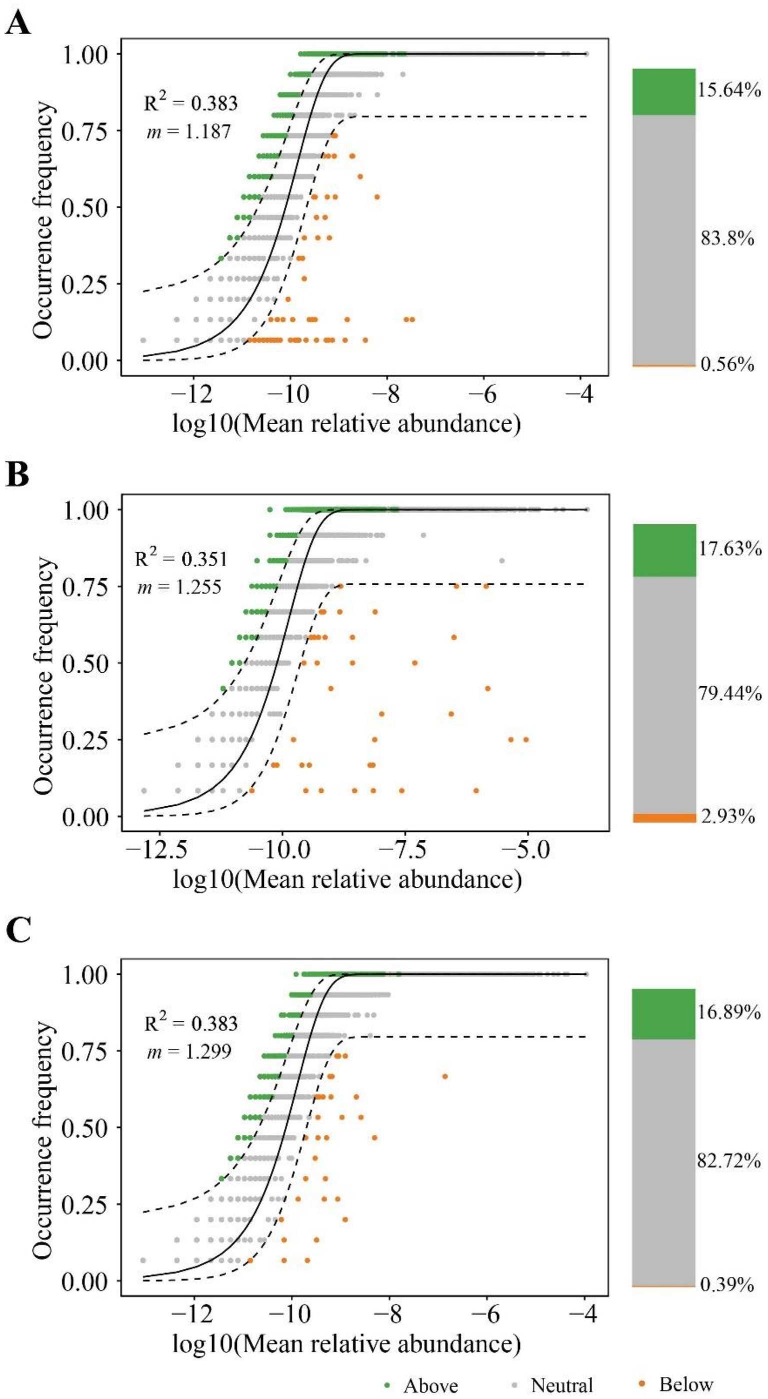

Fig. S4 NCM of August (A), September (B), and October (C).

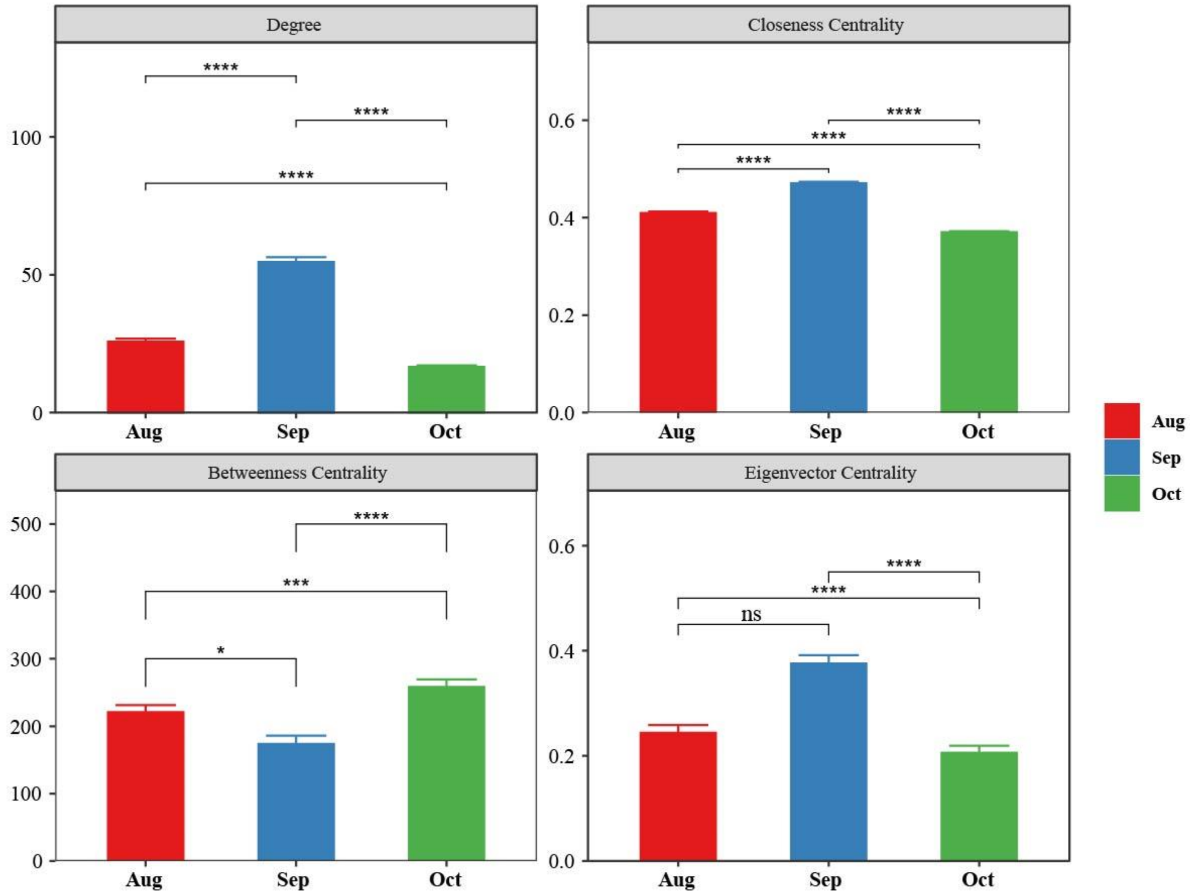

Fig. S5 Co-occurrence network of microbial OTUs based on node features of the network.

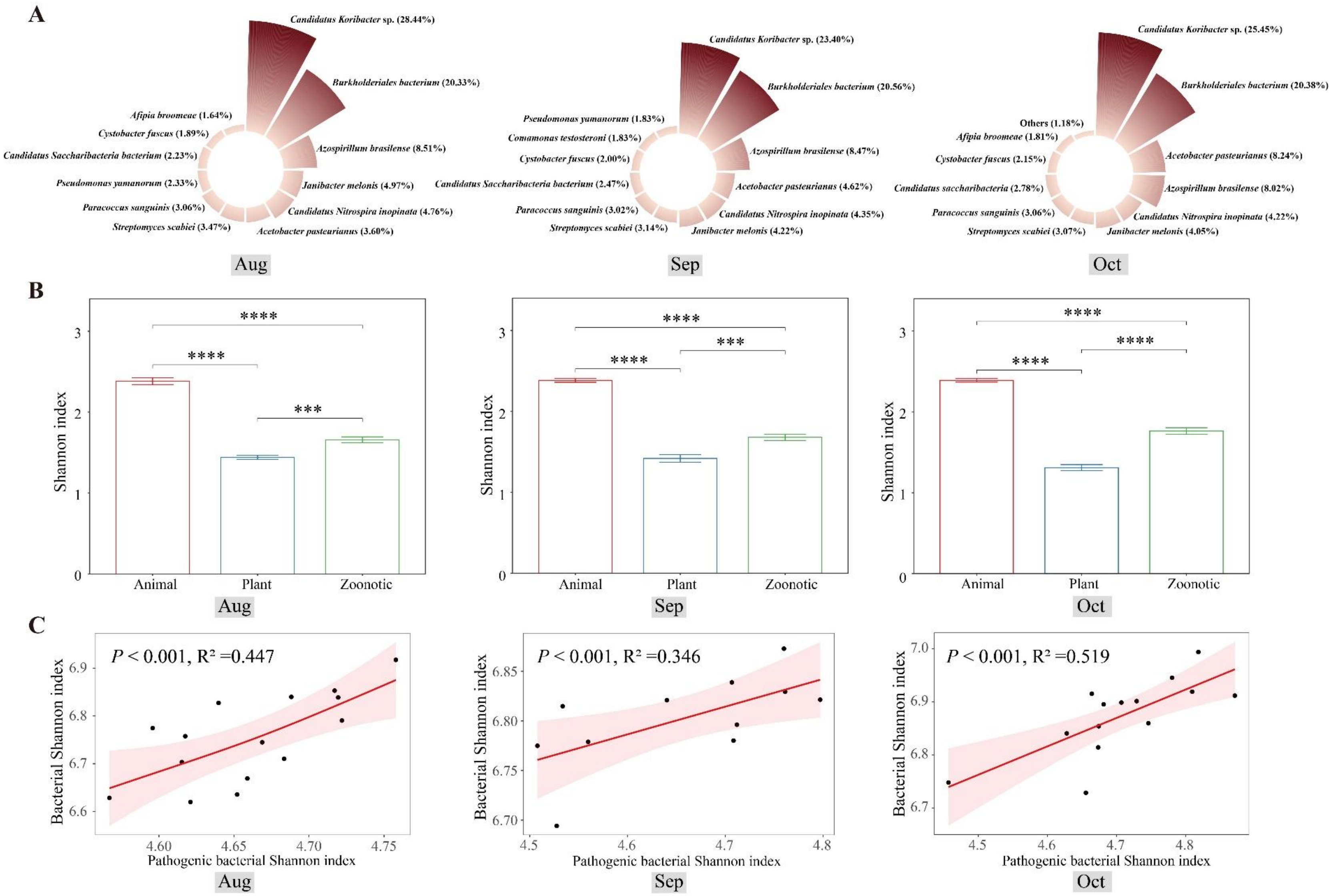

Fig. S6 Main species of soil pathogenic bacteria in the study area (A), Shannon index (B), and the relationship (C) shown by the generalized additive model (GAM) between pathogenic bacterial Shannon index and bacterial Shannon index in August, September, and October.

Table S1. The abundance of the top ten bacterial phyla in the soil rhizosphere of *Salix matsudana* for every week (Mean ± SE).

Phyla	EW0801	EW0808	EW0815	EW0822	EW0829	EW0905	EW0912	EW0919	EW0926	EW1003	EW1010	EW1017	EW1024	EW1031
Actinobacteriota	0.305±0.013^{a}	0.254±0.009c	0.254±0.020^{c}	0.262±0.018^{bc}	0.277±0.013^{abc}	0.275±0.016^{abc}	0.276±0.006^{abc}	0.259±0.011^{bc}	0.297±0.027^{ab}	0.255±0.009^{bc}	0.260±0.006^{bc}	0.270±0.009^{abc}	0.259±0.011^{bc}	0.272±0.014^{abc}
Proteobacteria	0.245±0.018^{ab}	0.212±0.011^{ab}	0.217±0.011^{ab}	0.216±0.008^{ab}	0.223±0.019^{ab}	0.236±0.023^{ab}	0.228±0.022^{ab}	0.225±0.022^{ab}	0.240±0.017^{ab}	0.197±0.010^{b}	0.210±0.011^{ab}	0.197±0.002^{b}	0.270±0.049^{a}	0.225±0.019^{ab}
Acidobacteriota	0.142±0.021^{bc}	0.219±0.014^{a}	0.187±0.014^{abc}	0.190±0.016^{ab}	0.171±0.034^{abc}	0.167±0.030^{abc}	0.172±0.029^{abc}	0.195±0.034^{ab}	0.119±0.033^{c}	0.213±0.014^{ab}	0.177±0.011^{abc}	0.183±0.005^{abc}	0.157±0.028^{abc}	0.160±0.029^{abc}
Chloroflexi	0.110±0.010^{ab}	0.114±0.006^{ab}	0.121±0.010^{a}	0.117±0.006^{ab}	0.105±0.010^{ab}	0.104±0.008^{ab}	0.105±0.008^{ab}	0.105±0.008^{ab}	0.090±0.022^{b}	0.120±0.004^{a}	0.125±0.007^{a}	0.123±0.005^{a}	0.099±0.013^{ab}	0.109±0.006^{ab}
Gemmatimonadota	0.035±0.002^{a}	0.030±0.004^{a}	0.032±0.002^{a}	0.029±0.001^{a}	0.030±0.001^{a}	0.030±0.000^{a}	0.031±0.002^{a}	0.030±0.002^{a}	0.028±0.005^{a}	0.030±0.002^{a}	0.033±0.000^{a}	0.031±0.001^{a}	0.030±0.004^{a}	0.033±0.001^{a}
Bacteroidota	0.026±0.000^{b}	0.022±0.003^{b}	0.023±0.000^{b}	0.026±0.001^{b}	0.031±0.006^{b}	0.025±0.000^{b}	0.029±0.001^{b}	0.029±0.000^{b}	0.081±0.054^{a}	0.024±0.003^{b}	0.029±0.005^{b}	0.026±0.002^{b}	0.033±0.001^{b}	0.026±0.000^{b}
Myxococcota	0.025±0.001^{bc}	0.022±0.001^{c}	0.026±0.000^{abc}	0.030±0.002^{abc}	0.030±0.002^{abc}	0.032±0.001^{ab}	0.033±0.002^{a}	0.028±0.003^{abc}	0.026±0.006^{abc}	0.026±0.002^{abc}	0.029±0.001^{abc}	0.030±0.001^{abc}	0.028±0.001^{abc}	0.030±0.002^{ab}
Methylomirabilota	0.020±0.003^{bc}	0.024±0.000^{abc}	0.030±0.001^{a}	0.023±0.006^{abc}	0.024±0.002^{abc}	0.026±0.001^{ab}	0.024±0.001^{abc}	0.023±0.003^{abc}	0.016±0.005^{c}	0.022±0.001^{abc}	0.024±0.001^{abc}	0.020±0.003^{bc}	0.017±0.002^{c}	0.022±0.002^{abc}
Firmicutes	0.016±0.001^{bc}	0.014±0.003^{bc}	0.021±0.000^{bc}	0.012±0.001^{c}	0.026±0.011^{ab}	0.017±0.005^{bc}	0.016±0.005^{bc}	0.014±0.002^{bc}	0.035±0.001^{a}	0.026±0.001^{ab}	0.024±0.003^{abc}	0.025±0.002^{abc}	0.023±0.001^{abc}	0.034±0.007^{a}
Planctomycetota	0.010±0.001^{c}	0.019±0.001^{ab}	0.018±0.002^{ab}	0.019±0.002^{ab}	0.016±0.002^{abc}	0.018±0.004^{abc}	0.015±0.003^{abc}	0.020±0.003^{a}	0.012±0.002^{bc}	0.019±0.000^{ab}	0.019±0.001^{ab}	0.020±0.001^{ab}	0.015±0.003^{abc}	0.018±0.003^{abc}

Table S2. The abundance of the top ten bacterial genera in the soil rhizosphere of *Salix matsudana* for every week (Mean ± SE).

Genera	EW0801	EW0808	EW0815	EW0822	EW0829	EW0905	EW0912	EW0919	EW0926	EW1003	EW1010	EW1017	EW1024	EW1031
Arthrobacter	0.022±0.003^{ab}	0.021±0.006^{ab}	0.020±0.004^{ab}	0.019±0.001^{ab}	0.019±0.004^{b}	0.017±0.005^{b}	0.013±0.002^{b}	0.014±0.004^{b}	0.044±0.029^{a}	0.017±0.001^{b}	0.017±0.002^{b}	0.019±0.003^{ab}	0.019±0.003^{ab}	0.021±0.008^{ab}
Gaiella	0.023±0.001^{a}	0.019±0.001^{a}	0.019±0.001^{a}	0.018±0.000^{a}	0.019±0.001^{a}	0.021±0.001^{a}	0.022±0.002^{a}	0.021±0.003^{a}	0.018±0.003^{a}	0.019±0.000^{a}	0.020±0.001^{a}	0.017±0.001^{a}	0.017±0.001^{a}	0.022±0.003^{a}
Nocardioides	0.020±0.005^{ab}	0.013±0.001^{ab}	0.012±0.001^{ab}	0.018±0.005^{ab}	0.022±0.006^{b}	0.020±0.004^{b}	0.017±0.001^{b}	0.015±0.001^{b}	0.016±0.004^{a}	0.015±0.001^{b}	0.014±0.001^{b}	0.017±0.001^{ab}	0.025±0.009^{ab}	0.018±0.002^{ab}
MND1	0.017±0.001^{a}	0.017±0.001^{a}	0.017±0.001^{a}	0.018±0.001^{a}	0.017±0.000^{a}	0.018±0.000^{a}	0.019±0.003^{a}	0.019±0.000^{a}	0.016±0.004^{a}	0.015±0.000^{a}	0.017±0.000^{a}	0.016±0.000^{a}	0.016±0.002^{a}	0.018±0.001^{a}
RB41	0.012±0.001^{a}	0.016±0.000^{a}	0.018±0.003^{a}	0.019±0.002^{a}	0.018±0.005^{a}	0.016±0.004^{a}	0.017±0.003^{a}	0.015±0.003^{a}	0.010±0.003^{a}	0.017±0.000^{a}	0.016±0.002^{a}	0.017±0.002^{a}	0.012±0.002^{a}	0.012±0.002^{a}
Bacillus	0.010±0.000^{def}	0.009±0.002^{ef}	0.014±0.000^{bcdef}	0.008±0.001^{f}	0.010±0.002^{def}	0.012±0.004^{bcdef}	0.011±0.004^{cdef}	0.009±0.002^{ef}	0.020±0.006^{ab}	0.019±0.001^{abcd}	0.018±0.002^{abcde}	0.019±0.002^{abc}	0.016±0.001^{bcdef}	0.027±0.005^{a}
Blastococcus	0.016±0.002^{a}	0.011±0.000^{b}	0.009±0.000^{b}	0.012±0.003^{ab}	0.013±0.000^{ab}	0.013±0.000^{ab}	0.013±0.001^{ab}	0.014±0.002^{ab}	0.013±0.002^{ab}	0.011±0.000^{b}	0.012±0.000^{ab}	0.013±0.000^{ab}	0.012±0.001^{ab}	0.012±0.001^{b}
Solirubrobacter	0.014±0.002^{a}	0.010±0.000^{ab}	0.012±0.002^{ab}	0.012±0.002^{ab}	0.011±0.000^{ab}	0.012±0.001^{ab}	0.012±0.001^{ab}	0.010±0.001^{ab}	0.009±0.002^{b}	0.010±0.000^{ab}	0.011±0.001^{ab}	0.013±0.002^{ab}	0.008±0.000^{b}	0.010±0.001^{ab}
Sphingomonas	0.012±0.002^{a}	0.006±0.000^{bc}	0.008±0.000^{bc}	0.007±0.000^{bc}	0.007±0.001^{bc}	0.006±0.001^{bc}	0.006±0.001^{bc}	0.009±0.000^{b}	0.007±0.000^{bc}	0.006±0.000^{c}	0.006±0.000^{bc}	0.006±0.000^{bc}	0.008±0.000^{bc}	0.007±0.000^{bc}
Streptomyces	0.008±0.000^{ab}	0.006±0.000^{b}	0.007±0.000^{b}	0.006±0.000^{b}	0.010±0.002^{a}	0.007±0.001^{b}	0.007±0.000^{ab}	0.007±0.000^{b}	0.006±0.001^{b}	0.007±0.000^{b}	0.006±0.000^{b}	0.006±0.000^{b}	0.006±0.000^{b}	0.008±0.001^{ab}

Table S3. Lists of keystone taxa in co-occurrence network of bacteria.

Month	Number of nodes	OTU ID	Zi	Pi	Types	Relative abundance	Category	Taxonomy
Aug	1	OTU1235	-0.982	0.625	Connectors	0.1202%	CRT	p_Proteobacteria, c_Alphaproteobacteria, o_Tistrellales f_Geminicoccaceae
	2	OTU1539	0.337	0.628	Connectors	0.0857%	CRT	p_Acidobacteriota, c_Vicinamibacteria, o_Subgroup_17
	3	OTU1825	1.514	0.649	Connectors	0.0794%	CRT	p_Acidobacteriota, c_Acidobacteriae, o_Bryobacterales, f_Bryobacteraceae, g_*Bryobacter*
	4	OTU1841	0.614	0.647	Connectors	0.4655%	MT	p_Unclassified Bacteria
	5	OTU1852	0.141	0.626	Connectors	0.3241%	MT	p_Chloroflexi, c_KD4-96
	6	OTU237	-0.387	0.631	Connectors	0.4139%	MT	p_Actinobacteriota, c_Actinobacteria, o_Streptomycetales, f_Streptomycetaceae, g_*Streptomyces*, s_*Streptomyces scabiei*
	7	OTU2451	-0.919	0.660	Connectors	0.0762%	CRT	p_Proteobacteria, c_Gammaproteobacteria, o_Burkholderiales, f_Sutterellaceae
	8	OTU2901	1.500	0.656	Connectors	0.0775%	CRT	p_Proteobacteria, c_Alphaproteobacteria, o_Rhizobiales, f_KF-JG30-B3
	9	OTU308	-0.919	0.693	Connectors	0.1105%	CRT	p_Methylomirabilota, c_Methylomirabilia, o_Rokubacteriales
	10	OTU3551	-1.036	0.667	Connectors	0.1373%	CRT	p_Proteobacteria, c_Alphaproteobacteria, o_Rhizobiales f_Rhizobiaceae, g_*Mesorhizobium*
	11	OTU3778	1.411	0.654	Connectors	0.3997%	MT	p_Actinobacteriota, c_Thermoleophilia, o_Solirubrobacterales, f_Solirubrobacteraceae, g_*Solirubrobacter*
	12	OTU3795	-0.448	0.659	Connectors	0.1448%	CRT	p_Actinobacteriota, c_Actinobacteria , o_Propionibacteriales, f_Nocardioidaceae, g_*Nocardioides*
	13	OTU3834	-1.036	0.678	Connectors	0.0896%	CRT	p_Chloroflexi, c_Chloroflexia, o_Thermomicrobiales, f_JG30-KF-CM45
	14	OTU3884	0.880	0.661	Connectors	0.1722%	CRT	p_Proteobacteria, c_Alphaproteobacteria, o_Rhizobiales, f_Beijerinckiaceae, g_*Microvirga*
	15	OTU4080	-0.644	0.663	Connectors	0.1791%	MT	p_Actinobacteriota, c_Actinobacteria , o_Propionibacteriales, f_Nocardioidaceae
	16	OTU439	-0.184	0.649	Connectors	0.2419%	MT	p_Actinobacteriota, c_Thermoleophilia, o_Gaiellales
	17	OTU4587	-1.036	0.642	Connectors	0.1852%	MT	p_Actinobacteriota, c_Acidimicrobiia, o_Microtrichales, f_Iamiaceae, g_*Iamia*
	18	OTU4669	0.146	0.632	Connectors	0.6420%	MT	p_Proteobacteria, c_Alphaproteobacteria, o_Azospirillales, f_Azospirillaceae, g_*Skermanella*
	19	OTU4725	-0.716	0.684	Connectors	0.3289%	MT	p_Methylomirabilota, c_Methylomirabilia, o_Rokubacteriales
	20	OTU4969	0.533	0.629	Connectors	0.2363%	MT	p_Actinobacteriota, c_Actinobacteria, o_Propionibacteriales, f_Nocardioidaceae, g_*Nocardioides*, s_*Nocardioides exalbidus*
	21	OTU4976	2.298	0.669	Connectors	0.1985%	CRT	p_Actinobacteriota, c_Actinobacteria, o_Propionibacteriales, f_Nocardioidaceae, g_*Nocardioides*

	22	OTU5397	-1.036	0.667	Connectors	0.1426%	CRT	p_Proteobacteria, c_Gammaproteobacteria, o_Xanthomonadales, f_Xanthomonadaceae, g_*Lysobacter*
	23	OTU5478	-1.248	0.656	Connectors	0.0719%	CRT	p_Chloroflexi, c_Chloroflexia, o_Thermomicrobiales, f_AKYG1722
	24	OTU5695	0.082	0.680	Connectors	0.1353%	CRT	p_Myxococcota, c_bacteriap25
	25	OTU5707	-0.450	0.663	Connectors	0.1202%	CRT	p_Acidobacteriota, c_Thermoanaerobaculia, o_Thermoanaerobaculales, f_Thermoanaerobaculaceae, g_Subgroup_10
	26	OTU591	-1.225	0.750	Connectors	0.8403%	CAT	p_Proteobacteria, c_Gammaproteobacteria, o_Burkholderiales, f_Nitrosomonadaceae, g_MND1
	27	OTU594	0.925	0.679	Connectors	1.2884%	CAT	p_Actinobacteriota, c_Actinobacteria, o_Frankiales, f_Geodermatophilaceae, g_*Blastococcus*
	28	OTU5943	-0.500	0.660	Connectors	0.1219%	CRT	p_Chloroflexi, c_Chloroflexia, o_Thermomicrobiales, f__JG30-KF-CM45
	29	OTU6093	0.000	0.720	Connectors	0.0798%	CRT	p_Proteobacteria, c_Gammaproteobacteria, o_Burkholderiales, f_Nitrosomonadaceae, g_MND1
	30	OTU6135	-0.251	0.675	Connectors	0.1459%	CRT	p_Proteobacteria, c_Alphaproteobacteria, o_Azospirillales, f_Azospirillaceae, g_*Skermanella*
	31	OTU6176	-0.982	0.653	Connectors	0.0762%	CRT	p_Chloroflexi, c_Dehalococcoidia, o_S085
	32	OTU6420	-1.036	0.656	Connectors	0.1073%	CRT	p_Proteobacteria, c_Gammaproteobacteria, o_Burkholderiales, f_Comamonadaceae
	33	OTU6791	-1.514	0.708	Connectors	0.0814%	CRT	p_Proteobacteria, c_Gammaproteobacteria, o_Xanthomonadales, f_Xanthomonadaceae, g_Lysobacter
	34	OTU7040	-1.248	0.625	Connectors	0.0971%	CRT	p_Firmicutes, c_Bacilli, o_Bacillales, f_Bacillaceae, g_*Bacillus*
	35	OTU7313	-1.232	0.722	Connectors	0.1966%	MT	p_Proteobacteria, c_Gammaproteobacteria, o_Xanthomonadales, f_Xanthomonadaceae, g_*Lysobacter*
	36	OTU914	-1.008	0.653	Connectors	0.1927%	MT	p_Actinobacteriota, c_Acidimicrobiia, o_Actinomarinales
Sep	1	OTU3712	-0.275	0.653	Connectors	0.0893%	CRT	p_Chloroflexi, c_JG30-KF-CM66
	2	OTU5005	-1.732	0.645	Connectors	0.0728%	CRT	p_Actinobacteriota, c_Acidimicrobiia, o_IMCC26256
	3	OTU6058	-1.257	0.642	Connectors	0.0718%	CRT	p_Actinobacteriota, c_Actinobacteria, o_Corynebacteriales, f_Nocardiaceae, g_*Nocardia*
Oct	1	OTU1119	1.581	0.694	Connectors	0.2115%	MT	p_Proteobacteria, c_Gammaproteobacteria, o_Burkholderiales, f_Nitrosomonadaceae, g_Ellin6067
	2	OTU1782	0.000	0.717	Connectors	0.0863%	CRT	p_Acidobacteriota, c_Vicinamibacteria, o_Vicinamibacterales
	3	OTU2391	-0.526	0.716	Connectors	0.0874%	CRT	p_Actinobacteriota, c_MB-A2-108
	4	OTU293	-0.069	0.712	Connectors	0.3241%	MT	p_Proteobacteria, c_Gammaproteobacteria, o_CCD24

5	OTU3001	3.069	0.000	Module hubs	0.2801%	MT	p_Actinobacteriota, c_Thermoleophilia, o_Gaiellales
6	OTU3346	-1.040	0.695	Connectors	0.3226%	MT	p_Proteobacteria, c_Gammaproteobacteria
7	OTU3736	-1.334	0.667	Connectors	0.0982%	CRT	p_Proteobacteria, c_Alphaproteobacteria, o_Reyranellales, f_Reyranellaceae, g_*Reyranella*
8	OTU3795	-1.241	0.642	Connectors	0.1437%	CRT	p_Actinobacteriota, c_Actinobacteria, o_Propionibacteriales, f_Nocardioidaceae, g__*Nocardioides*
9	OTU3977	1.092	0.695	Connectors	0.0770%	CRT	p_Actinobacteriota, c_Actinobacteria, o_Propionibacteriales, f_Nocardioidaceae, g_*Nocardioides*
10	OTU4251	-0.853	0.645	Connectors	0.2706%	MT	p_Chloroflexi, c_Chloroflexia, o_Thermomicrobiales, f_AKYG1722
11	OTU4269	-1.442	0.688	Connectors	0.0803%	CRT	p_Bacteroidota, c_Bacteroidia, o_Chitinophagales, f_Saprospiraceae
12	OTU4486	-0.035	0.645	Connectors	0.0991%	CRT	p_Acidobacteriota, c_Vicinamibacteria, o_Vicinamibacterales, f_Vicinamibacteraceae
13	OTU4587	-0.526	0.765	Connectors	0.1936%	MT	p_Actinobacteriota, c_Acidimicrobiia, o_Microtrichales, f_Iamiaceae, g_*Iamia*
14	OTU4669	-0.236	0.676	Connectors	0.6463%	MT	p_Proteobacteria, c_Alphaproteobacteria, o_Azospirillales, f_Azospirillaceae, g_*Skermanella*
15	OTU4756	-1.167	0.625	Connectors	0.1321%	CRT	p_Acidobacteriota, c_Vicinamibacteria, o_Vicinamibacterales
16	OTU5486	-0.236	0.670	Connectors	0.0779%	CRT	p_Acidobacteriota, c_Thermoanaerobaculia, o_Thermoanaerobaculales, f_Thermoanaerobaculaceae, g_Subgroup_10
17	OTU5510	-0.789	0.628	Connectors	0.1068%	CRT	p_Acidobacteriota, c_Vicinamibacteria, o_Vicinamibacterales, f_Vicinamibacteraceae
18	OTU5513	-0.695	0.635	Connectors	0.2909%	MT	p_Proteobacteria, c_Alphaproteobacteria, o_Rhizobiales, f_Xanthobacteraceae
19	OTU5695	-0.035	0.635	Connectors	0.1286%	CRT	p_Myxococcota, c_bacteriap25
20	OTU594	-1.040	0.645	Connectors	1.2290%	CAT	p_Actinobacteriota, c_Actinobacteria, o_Frankiales, f_Geodermatophilaceae, g_Blastococcus
21	OTU5943	-1.481	0.625	Connectors	0.0984%	CRT	p_Chloroflexi, c_Chloroflexia, o_Thermomicrobiales, f_JG30-KF-CM45
22	OTU5959	-0.121	0.639	Connectors	0.1720%	CRT	p_Acidobacteriota, c_Vicinamibacteria, o_Vicinamibacterales, f_Vicinamibacteraceae
23	OTU6135	-1.010	0.653	Connectors	0.1524%	MT	p_Proteobacteria, c_Alphaproteobacteria. o_Azospirillales, f_Azospirillaceae, g_Skermanella
24	OTU6395	2.778	0.077	Module hubs	0.4236%	MT	p_Actinobacteriota, c_Actinobacteria, o_Propionibacteriales, f_Nocardioidaceae, g_*Marmoricola*
25	OTU6585	0.000	0.735	Connectors	0.8572%	CAT	p_Chloroflexi, c_Chloroflexia, o_Thermomicrobiales, f_JG30-KF-CM45
26	OTU6637	-1.010	0.700	Connectors	0.2678%	MT	p_Gemmatimonadota, c_Gemmatimonadetes, o_Gemmatimonadales, f_Gemmatimonadaceae
27	OTU666	-1.643	0.640	Connectors	0.0898%	CRT	p_Gemmatimonadota, c_Gemmatimonadetes, o_Gemmatimonadales, f_Gemmatimonadaceae
28	OTU6695	0.000	0.667	Connectors	0.0818%	CRT	p_Proteobacteria, c_Alphaproteobacteria, o_Rhizobiales, f_Hyphomicrobiaceae,

							g_*Pedomicrobium*
29	OTU7193	-0.853	0.675	Connectors	0.0786%	CRT	p_Chloroflexi, c_Chloroflexia, o_Chloroflexales, f_Roseiflexaceae
30	OTU7213	0.166	0.620	Connectors	0.1221%	CRT	p_Actinobacteriota, c_Actinobacteria, o_Propionibacteriales, f_Nocardioidaceae, g_Nocardioides
31	OTU7216	-1.581	0.708	Connectors	0.1038%	CRT	p_Actinobacteriota, c_Actinobacteria, o_Micromonosporales, f_Micromonosporaceae, g_*Rhizocola*
32	OTU7520	-0.930	0.722	Connectors	0.0723%	CRT	p_Proteobacteria, c_Alphaproteobacteria, o_Sphingomonadales, f_Sphingomonadaceae, g_*Novosphingobium*
33	OTU839	-0.121	0.653	Connectors	0.2186%	MT	p_Actinobacteriota, c_Actinobacteria, o_Micrococcales, f_Microbacteriaceae, g_*Agromyces*
34	OTU909	-0.696	0.711	Connectors	0.0740%	CRT	p_Proteobacteria, c_Alphaproteobacteria, o_Rhizobiales, f_Hyphomicrobiaceae, g_*Hyphomicrobium*
